# Why so many unknown genes? Partitioning orphans from a representative transcriptome of the lone star tick *Amblyomma americanum*

**DOI:** 10.1186/1471-2164-14-135

**Published:** 2013-02-27

**Authors:** Amanda K Gibson, Zach Smith, Clay Fuqua, Keith Clay, John K Colbourne

**Affiliations:** 1 Department of Biology, Indiana University, Bloomington, IN, 47405, USA; 2 The Center for Genomics and Bioinformatics, Indiana University, Bloomington, IN, 47405, USA; 3 Current address: School of Biosciences, University of Birmingham, Birmingham, B15 2TT, United Kingdom

**Keywords:** *Amblyomma americanum*, Chelicerata, EST (Expressed Sequence Tags), Microarray, Orphan genes, Taxonomic isolation, Tick, Transcriptome

## Abstract

**Background:**

Genomic resources within the phylum Arthropoda are largely limited to the true insects but are beginning to include unexplored subphyla, such as the Crustacea and Chelicerata. Investigations of these understudied taxa uncover high frequencies of orphan genes, which lack detectable sequence homology to genes in pre-existing databases. The ticks (Acari: Chelicerata) are one such understudied taxon for which genomic resources are urgently needed. Ticks are obligate blood-feeders that vector major diseases of humans, domesticated animals, and wildlife. In analyzing a transcriptome of the lone star tick *Amblyomma americanum*, one of the most abundant disease vectors in the United States, we find a high representation of unannotated sequences. We apply a general framework for quantifying the origin and true representation of unannotated sequences in a dataset and for evaluating the biological significance of orphan genes.

**Results:**

Expressed sequence tags (ESTs) were derived from different life stages and populations of *A. americanum* and combined with ESTs available from GenBank to produce 14,310 ESTs, over twice the number previously available. The vast majority (71%) has no sequence homology to proteins archived in UniProtKB. We show that poor sequence or assembly quality is not a major contributor to this high representation by orphan genes. Moreover, most unannotated sequences are functional: a microarray experiment demonstrates that 59% of functional ESTs are unannotated. Lastly, we attempt to further annotate our EST dataset using genomic datasets from other members of the Acari, including *Ixodes scapularis*, four other tick species and the mite *Tetranychus urticae*. We find low homology with these species, consistent with significant divergence within this subclass.

**Conclusions:**

We conclude that the abundance of orphan genes in *A. americanum* likely results from 1) taxonomic isolation stemming from divergence within the tick lineage and limited genomic resources for ticks and 2) lineage-specific genes needing functional genomic studies to evaluate their association with the unique biology of ticks. The EST sequences described here will contribute substantially to the development of tick genomics. Moreover, the framework provided for the evaluation of orphan genes can guide analyses of future transcriptome sequencing projects.

## Background

Genome sequencing efforts focused upon the phylum Arthropoda have grown enormously with advances in genomics and bioinformatics. As of May, 2012, the National Center for Biotechnology Information (NCBI) reported 222 arthropod genomes as assembled or in progress. Importantly, 82% of these projects are for true insects (Hexapoda: Insecta). Genomic resources for the remainder of the arthropod phylum are far more limited: 24 projects are reported for crustaceans, one for myriapoda, and 16 for chelicerates. The subphyla have divergent evolutionary histories exceeding 500 million years [1]. Broadening the genome survey across this phylogenetic distance contributes to the discovery of lineage-specific genes [2-4], making the sources of orphan genes a particularly relevant question for these taxa [5,6].

Lack of homology to genomic databases prevents the putative assignment of function to orphans, which typically represent at least 10% of an organism’s gene set [3,7]. They are commonly attributed to adaptations associated with a taxon’s unique biology [3]. This argument was most recently advocated by researchers of the *Daphnia pulex* genome, in which a remarkable 36% of genes showed no homology to other datasets [5]. There are, however, numerous potential sources for orphan gene sequences that must be thoroughly investigated in light of their high representation in sequenced genomes and transcriptomes. Poor quality sequence and/or assembly are the least interesting and arguably most likely sources for unassigned sequences. Moreover, genes without homology to other datasets may be non-functional [3,8-13]. Taxonomic isolation among representative lineages in genome databases can also contribute to lack of homology. For instance, as the first crustacean and chelicerate genomes with annotated genomes, the proportion of orphans in *D. pulex* and in *Tetranychus urticae* far exceeds that for closely-related but more heavily-sampled insect genomes [5-7].

In consideration of orphan genes, transcriptome projects serve as an important complement to whole-genome sequencing. They provide a more rapid and less expensive approach to obtaining gene sequences. In addition, transcriptome sequencing projects typically focus exclusively upon protein-coding regions. These are translated to amino acid sequences, which are more likely to be conserved [14-16]. Focusing upon conserved sequences favors identification of true orphan genes. Finally, transcriptomes are an effective proxy for estimating gene diversity and sampling orphan genes when other genomic data are limited. This is contingent upon having a sufficient number of expressed sequence tags (ESTs) that are enriched for full-length transcripts, normalized to sample rare mRNA, and sampled from biologically variable pools of RNA to obtain transcripts associated with diverse tissue types and biological processes [5,15,17,18].

The arthropod subphylum Chelicerata includes scorpions, horseshoe crabs, spiders, mites, and ticks. These lineages are more diverse than Crustacea and equally understudied. The chelicerate subclass Acari comprises the tick and mite lineages. Within the Acari, draft genomes of *Tetranychus urticae*, the two-spotted spider mite [6], and *Ixodes scapularis*, the black-legged deer tick [19,20] are available, with that of *Rhipicephalus microplus*, the southern cattle tick, in progress [21,22]. The need for more comprehensive genomic and transcriptomic data within the Acari is pressing given that many species are obligate blood-feeders that vector human and animal pathogens, including typhus, Lyme disease, Rocky Mountain Spotted Fever, and ehrlichiosis [23]. More data from blood-feeding chelicerates would allow comparison within ticks and with blood-feeding insects for identification of shared pathways to be exploited in control efforts. Indeed, numerous transcriptome projects targeting the salivary glands of at least 12 tick species have implicated several gene families as central to blood-feeding [24-35].

The lone star tick, *Amblyomma americanum*, is one of the most abundant vectors of zoonotic pathogens in the United States [36-38]. White-tailed deer are key hosts of *A. americanum*, and their ongoing expansion into suburban areas has increased tick-human interactions [36-38]. Lone star tick bites are associated with many diseases including human monocytic ehrlichiosis [39], southern tick-associated rash illness [40,41], tularemia [42], several pathogenic *Rickettsia*[36-38,43], and perhaps the recently discovered Heartland Virus [44]. As of September 2012, only 6,502 ESTs were available for *A. americanum* on GenBank [45], derived primarily from specific analysis of gene expression associated with tick salivary glands and blood-feeding [32,46]. A whole-organism transcriptome would complement these previously available sequences by increasing gene number and diversity.

Here, we present a comprehensive study of a normalized EST library for *A. americanum* enriched for unique, non-redundant transcripts. This library more than doubles the number of sequences previously available for this species. It represents a compilation of sequences from five life stages from a laboratory colony (i.e. larva, nymph, adult male, adult female, engorged female) and from a cohort of ticks collected from the wild. This approach reveals a large number of genes lacking homology to existing tick and other arthropod genomic datasets. We also outline a framework for evaluating orphan genes, with the aim to distinguish the primary sources of non-homology. Our results argue for a greater recognition and critical assessment of lineage-specific genes, notably in ticks and other understudied taxa.

## Results/Discussion

## EST sampling and sequencing

cDNA libraries were constructed from five developmental stages (larvae, nymph, adult male, adult female, engorged female) of *Amblyomma americanum*, reared under laboratory conditions. An additional library was constructed from a wild-collected population of 50 adult males and 50 adult females. Each library was normalized to reduce redundancy and improve gene discovery, particularly of rare transcripts. Details of preparation and single-pass Sanger-sequencing are provided in the Methods section. The numbers of cloned cDNA inserts derived from each of the libraries are presented in Additional file 1: Table S1.

## Assembly

The ESTPiper analysis tool [47] was applied for base calling and data cleaning, resulting in removal of 4,866 low-quality reads. The CAP3 [48] component of the ESTPiper sequence analysis tool was used to assemble the remaining 15,390 high quality sequences. The assembly yielded 12,319 unique sequences, comprising 10,443 singletons and 1,876 contigs, of which 86% were assemblies of two to three sequences (Additional file 1: Table S2a). Average redundancy was therefore estimated at 12%, with average gene discovery accordingly estimated at 88%.

An additional 6,502 *A. americanum* ESTs were available through GenBank. To enhance the size of our dataset, the 12,319 unique sequences generated from our six normalized libraries were secondarily assembled with these GenBank sequences using the CAP3 component of the ESTPiper. This nested assembly procedure allowed a primary, high-quality assembly of our six normalized libraries, followed by a secondary assembly with the potentially more variable and polymorphic sequences from GenBank. In doing so, we aimed to obtain a greater representation of gene transcripts. The secondary assembly produced 14,310 unique sequences, comprising 11,580 singletons, and 2,730 contigs, of which 76% were assemblies of two to three sequences (Additional file 1: Table S2b). Average redundancy was estimated at 14% and average gene discovery at 86%. The 6,502 GenBank sequences were therefore on average more likely to be redundant than our initial assembly of the six normalized libraries.

The distribution of ESTs across the six individual libraries (larvae, nymph, adult male, adult female, engorged female, and wild-collected) allowed examination of variation in expressed genes across developmental stage. This analysis served to identify transcripts that are expressed preferentially in a specific life stage or population. The results are discussed in detail in the Additional file 2.

## Annotation

**Figure 1:**
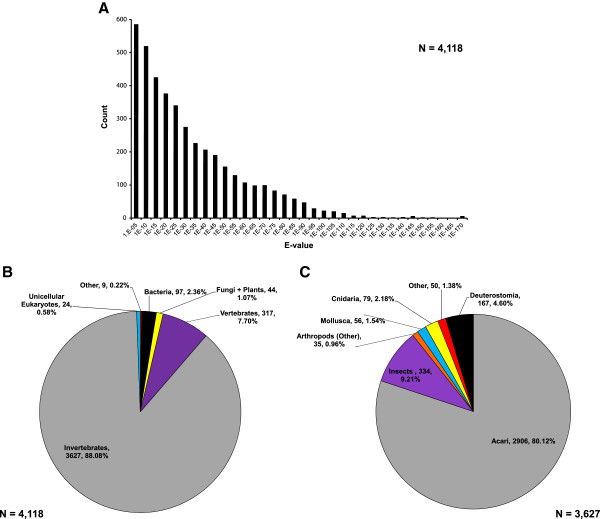
**Summary of the UniProtKB annotation of the secondary assembly of the *Amblyomma americanum *EST library.** (**A**) The e-value distribution of all annotated returns and the taxonomic distribution of (**B**) all annotated returns n = 4,118 and (**C**) returns annotated as invertebrate n = 3,627.

The assembled sequences were processed using the ESTPiper for annotation against the UniProtKB protein database (date: August 7, 2011) [49]. Of the 14,310 sequences, 4,118 (29%) matched at least one known protein. All BLAST searches reported in this study limit returns to an e-value cutoff of 1 × 10^-5^ and a minimum length of 33 aligned amino acid residues. Among the 2,730 assembled contigs, 1,243 (46%) matched at least one known protein (Table 1). For all sequences, the distribution of e-value scores indicated that our search against the UniProtKB database provided predominantly strong matches: 86% had scores ≤ 1 × 10^-10^ and 24% had scores ≤ 1 × 10^-50^ (Figure 1A). For protein matches against the 1,243 annotated contigs, the distribution of e-value scores demonstrated a similar pattern: 80% had scores ≤ 1 × 10^-10^ and 32% had scores ≤ 1 × 10^-50^. Therefore, the number of ESTs in our dataset with a protein match is proportionally low, but the returned matches were overall significant.

**Table 1:** **Summary of the annotation of the secondary *Amblyomma americanum *EST assembly**

	
**A. UniProtKB Annotation**	**Number**
Input sequences	14,310
Short (< 33 amino acids) returns	171
Weak (e-value > 1E-5) returns	145
Total unannotated returns	10,192
Annotated singletons	2,875
Annotated contigs	1,243
Total annotated sequences	4,118
**B. Peptide Database: Species**	**No. matches**
*Ixodes scapularis*	2,842
*Tetranychus urticae*	143
*Daphnia pulex*	208
*Pediculus humanus*	107
*Acyrthosiphon pisum*	225
*Apis mellifera*	140
*Tribolium castaneum*	218
*Bombyx mori*	28
*Aedes aegypti*	49
*Anopheles gambiae*	39
*Culex quinquefasciatus*	46
*Drosophila melanogaster*	52
**Total Hits**	4,099 (28.6%)

Statistics given for annotation against (A) the UniProtKB protein database and (B) a collection of arthropod datasets. A BLAST search of *A. americanum* ESTs against a compilation of 12 arthropod peptide datasets was conducted. Species are arranged to reflect increasing phylogenetic distance from *A. americanum*.

For the 29% of EST sequences matching a known protein, a survey of the distribution of taxonomic domains for returned comparative protein alignments revealed that the vast majority (88%) of *A. americanum* sequences matched proteins derived from invertebrates (Figure 1B). More specifically, 71% matched proteins from Acari lineages, and 9% matched proteins from other arthropods, predominantly insects (Figure 1C). In total, 4,015 of the annotated returns matched eukaryotic proteins, while six matched proteins derived from viruses and 97 from bacteria. The majority of these bacterial annotations (n = 76, 78%) were identified as members of the gram-negative γ-proteobacterial family Coxiellaceae. These sequences likely derive from the *Coxiella sp.* endosymbiont of *A. americanum* and are discussed in detail in the Additional file 2.

To investigate gene conservation more broadly across the arthropod phylum, we conducted a BLAST search of our *A. americanum* EST library against the predicted peptides of nine insect, one crustacean, and two chelicerate species with quality genome annotations. Of the 14,310 assembled EST sequences, 4,099 (29%) matched at least one arthropod peptide (Table 1). This proportion is nearly identical to the proportion of EST sequences matching proteins in UniProtKB. As expected, the vast majority (n = 2,842, 69%) of these matches were to *I. scapularis* peptides. The next most highly represented taxonomic groups were the aphid *Acyrthoshipon pisum* at 225 matches, the flour beetle *Tribolium castaneum* at 218, and the crustacean *D. pulex* at 208. The least represented groups were the three mosquito species *Aedes aegypti* at 49 matches, *Culex quinquefasciatus* at 46, and *Anopheles gambiae* at 39, and the silk moth *Bombyx mori* at 28 (Table 1). Low sequence homology was also observed between *A. americanum* and a fellow member of the Acari, *T. urticae*, indicating significant diversification within this subclass.

## Why are there so many unknown sequences in the Amblyomma americanum transcriptome?

In the genomic comparisons reported thus far, no more than 29% of the *A. americanum* transcriptome match genes that were annotated in other organisms. Therefore, at least 71% of the ESTs lack homology to pre-existing datasets based upon this preliminary annotation. We present here four potential sources of these unknown genes and assess their validity as explanations for the high representation observed in the *A. americanum* transcriptome. Because high proportions of unknown genes are commonly observed when annotating transcriptomes for a wide diversity of taxa [13,50-52], this framework is designed to help guide the reporting of unknown genes in future transcriptome projects.

## Hypothesis 1: the sequences are low quality

**Figure 2:**
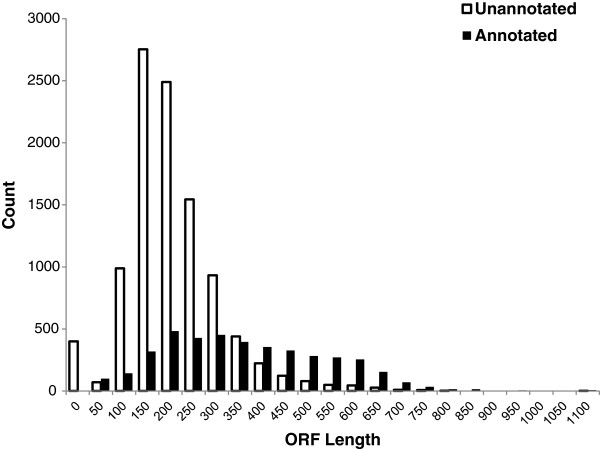
**Distribution of the nucleotide lengths of open reading frames for annotated and unannotated EST sequences.** Annotated ESTs are shown in black and unannotated in white. Open-reading frame lengths were predicted using the OrfPredictor component of the Transcriptome Analysis Pipeline of the Integrative Services for Genomic Analysis (ISGA) at Indiana University’s Center for Genomics and Bioinformatics. Annotation was determined by a BLAST search of the *A. americanum* EST library against the UniProtKB protein database.

Unannotated ESTs may be attributed to differences in sequence quality, as measured by length of predicted open reading frames (ORFs), presence of start codons, EST nucleotide length, and GC-content [3,8-13]. The most striking contrast between unannotated and annotated sequences from our study was ORF length. The mean ORF length, given by OrfPredictor, was significantly shorter for unannotated EST sequences as compared to annotated ESTs (t = 53.19; p < 0.0001, df = 5215) (Table 2, Figure 2). Likewise, the average length of annotated contigs was longer than that of unannotated contigs. Additionally, the mean nucleotide length of annotated ESTs was significantly larger than that of unannotated ESTs (t = 15.27; p < 0.0001, df = 7824). Accordingly, the mean nucleotide length of annotated contigs was longer than that of unannotated contigs. OrfPredictor also did not predict a start codon for 4% of the 10,192 unannotated sequences and, more specifically, for 3% of the 1,487 unannotated contigs. Lastly, the mean GC-content was significantly higher for annotated ESTs relative to unannotated ones (t = 41.41; p < 0.0001, df = 7917). Likewise, annotated contigs had a higher GC-content than unannotated contigs.

**Table 2:** Comparison of quality measures for annotated and unannotated ESTs

		**Annotated**	**Unannotated**
Mean ORF length (nts)	Sequences	339.9	183.4
	Contigs	376.7	213.1
Mean length (nts)	Sequences	594.9	536.0
	Contigs	746.5	735.8
Number w/o start codon	Sequences	0	398
	Contigs	0	38
Mean GC-content	Sequences	50	45.2
	Contigs	49.7	45.1

Comparisons are included for mean open-reading frame (ORF) length, mean nucleotide length, number of ESTs lacking a start codon, and mean GC-content. Statistics were obtained using OrfPredictor (ORF length and start codons) and Geneious software (nucleotide length and GC-content).

From this analysis of sequence quality, we can reject those sequences without start codons, which represent 4% of our 10,192 unannotated ESTs. The remaining 9,749 unannotated ESTs represent 70% of the EST library. Other quality metrics are less conducive to such definitive cut-offs. In spite of their statistical difference, the annotated and unannotated EST sets do not differ dramatically in measured quality scores (Table 2). Even for the most divergent metric, ORF length, the distributions of annotated and unannotated ESTs overlap substantially (Figure 2). Previous studies have consistently shown that sequence quality measures differ between annotated and unannotated sequences, with unannotated sequences shorter on average [3,8-13]. Length disparity was initially interpreted as the result of overassignment of ORFs, with short sequences erroneously assigned as protein-coding regions [11,12]. More recent studies, however, have confirmed that many orphan sequences are indeed protein-coding [5,10,53,54] and that they often encode shorter peptides than annotated sequences [3,10,55]. The longer reads of Sanger sequencing and the focus on expressed sequences in this study reduce the risk that unannotated sequences result from overassignment of short ORFs. Functional analysis is required, however, to fully account for the contribution of poor sequence quality to the proportion of unannotated sequences in our dataset (see Hypothesis 3).

## Hypothesis 2: the assembly is low quality

Low assembly quality could contribute to the detection of unannotated sequences if under-assembly occurred and reads lacking homology to other organisms preferentially failed to assemble. Our study benefits from the relatively longer reads of Sanger sequencing. These provide a greater number of potential sequence overlaps during the assembly processes in comparison to the shorter reads of next-generation sequencers. Nevertheless, the rate of assembly failure can be estimated by calculating the percentage of conserved single-copy arthropod genes that are present in multiple copies in the *A. americanum* transcriptome. euGenes/Arthropods [7] reports gene families shared among 14 arthropod families, as well as copy number in each species. Gene family (ARP2) IDs for *A. americanum* ESTs were identified through searching for sequence homology against the *I. scapularis* peptide database. Therefore, the following results only pertain to those ESTs that matched *I. scapularis* peptides. The ARP2 dataset lists 1,115 gene families that are composed of conserved single-copy orthologs across all 14 arthropod species. Of these, 415 have a match in the *A. americanum* EST library. A subset of 86 gene families (21%) contained more than one copy in our *A. americanum* gene set, likely representing assembly failures rather than duplications in the *A. americanum* lineage. The majority of these single-copy gene families contain two sequences/contigs in *A. americanum*, though 12 families had three reported copies, two families had four, and two families had five gene sequences. Therefore, 194 unique sequences in our EST assembly in fact represent only 86 single-copy genes (44%). The proportion of single-copy gene families present in multiple copies (21%) multiplied by the proportion of true single-copy genes generated by assembly failures (44%) suggest an assembly failure rate of 9%. The total number of unique ESTs in our assembly may thereby be reduced by approximately 1,315 ESTs, from 14,310 to 12,995. This analysis indicates that under-assembly has slightly affected the estimate of the number of non-redundant genes in the *A. americanum* EST library.

The above analysis alone cannot indicate if under-assembly has preferentially failed to assemble unannotated sequences. This might occur, for example, if shorter sequences, which are on average less likely to be annotated, are also less likely to assemble. To specifically address under-assembly of unannotated genes, the assembly process was modified to measure the contribution of assembly quality to the percentage of unannotated sequences. The 12,319 original and 6,502 GenBank ESTs were assembled, without nesting, using Newbler (454 Life Sciences) and updated CAP3 assemblers. Annotation of these two assemblies produced the same results as the previous nested assembly, suggesting that representation of unannotated sequences was not biased by our choice of assembly approach.

We emphasize that our results here do not imply that sequence and assembly quality do not contribute to the detection of unannotated sequences. Rather, we find that, in our dataset, we are unable to detect a strong signal of either low sequence or assembly quality in relation to our estimation of the representation by unannotated sequences.

## Hypothesis 3: these sequences do not represent functional genes

Another explanation for a high proportion of unknown genes is that these sequences do not in fact represent functional genes. Unannotated ESTs may include intronic or untranscribed sequences, due to cloning errors, or sequences with shifted reading frames, due to sequencing errors [8,56]. To explore this possibility, we developed microarrays to measure the expression level of 13,962 of the 14,310 *A. americanum* ESTs across 12 different conditions, distinguished by life stage and environment. A total of 5,000 sequences showed no evidence of expression in 11 or 12 conditions by virtue of falling below the detection threshold of a 0.5% false discovery rate. This threshold was set by the signal level distribution of 11,657 markov-modeled random sequence probes. Therefore, 8,962 (64%) of ESTs were identified as functional, based upon detectable levels of condition-dependent transcription (Additional file 3: Figure S1). Of these, 3,710 (41%) matched proteins or sequences from UniProtKB and/or four *I. scapularis* genomic datasets (see Hypothesis 4) (Table 3). This reveals that 41% of functional *A. americanum* ESTs are annotated, which represents a slight improvement in annotation from the proportions estimated prior to discounting of non-functional ESTs. This is likely a conservative estimate. Many ESTs, in particular unannotated ones [10], may be expressed under limited environmental conditions that are not represented by this microarray experiment, thus leading to their classification as non-functional. Importantly, this conservative approach also increases the probability of excluding non-protein-coding transcripts with low or limited expression. These may include processed pseudogenes or mRNA with disrupted reading frames that can contribute to detection of unannotated sequences [56,57]. We conclude from our microarray experiment that there remains in this EST dataset a large proportion of functional genes lacking sequence similarity to genes in annotated genome databases.

**Table 3:** **Summary of results from microarray validation of functionality of ESTs of *Amblyomma americanum***

	**Number**
Total ESTs matching to microarray probes	13,962
ESTs retained: expression above 0.5% FDR threshold	8,962 (64.2%)
Retained ESTs with UniProtKB annotation	3,105 (34.6%)
Retained ESTs with *I. scapularis* match	3,623 (40.4%)
Total retained ESTs with annotation	3,710 (41.3%)

A subset of ESTs were matched to microarray probes and expression was measured under 12 different conditions. Sequences with expression that fell below a 0.5% false discovery rate in 11 or 12 of these conditions were rejected. The remaining ESTs were characterized as annotated or unannotated according to matches to the UniProtKB protein database and to four *I. scapularis* genomic databases.

## Hypothesis 4: taxonomic isolation of A. americanum contributes to detection of unknown sequences

## *Ixodes scapularis*

The availability of a draft genome sequence assembly and annotation for *I. scapularis* allowed investigation of gene conservation across the Metastriata (*Amblyomma)* and Prostriata (*Ixodes)* tick groups. These Ixodidae tick lineages are estimated to have shared a most recent common ancestor in the Triassic, approximately 241 mya [58,59]. We predicted that the percent matching between *A. americanum* ESTs and *I. scapularis* genomic datasets would be substantially higher than the percent matching to the UniProtKB database, because of shared ancestry of Ixodidae (hard tick) species. Four different datasets were used here to represent the *I. scapularis* genome: the EST sequences, predicted peptides, assembled contigs, and singletons. Homology of the *A. americanum* ESTs to these *I. scapularis* datasets ranged from 16% to 29%, a surprisingly low percentage that does not differ notably from the percentage of sequences annotated through UniProtKB (Table 4; Additional file 3: Figure S2). Accounting for redundant matches, the four BLAST searches against these *I. scapularis* datasets returned a total of 5,483 matches, representing 38% of the *A. americanum* ESTs. Those sequences with matches to proteins through UniProtKB were far more likely to have a match to *I. scapularis*: for each of the BLAST searches of *A. americanum* ESTs against the *I. scapularis* datasets, 73% or greater of returns also had a match in the UniProtKB database (Table 4). Therefore, the majority of *A. americanum* ESTs was found to have no match to UniProtKB proteins and no homology to *I. scapularis* genome, peptide, or EST sequences. This result supports a substantial degree of divergence between *Ixodes* and *Amblyomma* sequences. Thus unannotated ESTs in the *A. americanum* library may be unique to the *Amblyomma* or Metastriata lineages of the hard ticks*.*

**Table 4:** **Summary of BLAST searches of *Amblyomma americanum *against *Ixodes scapularis***

		**UniProtKB**		***A. americanum *ESTs**
**A. Datasets for *I. scapularis***		**Annotation**	**No Annotation**	**Totals**
**ESTs**	*Match*	2,997	457	3,454 (24.1%)
	*No Match*	1,121	9,735	10,856
**Predicted Peptides**	*Match*	3,413	179	3,592 (25.1%)
	*No Match*	705	10,013	10,718
**Contigs**	*Match*	1,724	642	2,365 (16.5%)
	*No Match*	2,394	9,550	11,944
**Singletons**	*Match*	3,279	845	4124 (28.8%)
	*No Match*	839	9,347	10,186
		***I. scapularis *ESTs**		
**B. Datasets for *I. scapularis***		**Totals**		
**Predicted Peptides**	*Match*	128,738 (66.2%)		
	*No Match*	70,722		
**Contigs**	*Match*	72,465 (37.3%)		
	*No Match*	121,995		
**Singletons**	*Match*	105,183 (54.1%)		
	*No Match*	89,277		

(A) *Amblyomma americanum* EST sequences matching *Ixodes scapularis* genomic datasets and (B) *I. scapularis* EST sequences matching other *I. scapularis* datasets. *Ixodes scapularis* datasets include ESTs, predicted peptides, singletons, and assembled contigs. (A) *Amblyomma americanum* results are categorized according to UniProtKB annotation status (annotation, no annotation) and *I. scapularis* match status (match, no match). The proportion of the *A. americanum* ESTs with a match is provided in parentheses for each *I. scapularis* dataset. (B) BLAST searches of *I. scapularis* ESTs (N = 194,460) against the three other *I. scapularis* datasets were then conducted to evaluate the quality of these datasets. These are classified according to match status. The proportion of *I. scapularis* ESTs with a match is provided in parentheses.

Another potential explanation for the low percent matching between *A. americanum* and *I. scapularis* may be the state of the *I. scapularis* genome. The *I. scapularis* genome is large (2,100 Mb) and contains a very high proportion of repetitive DNA (70%) [60,61]. As a result, the current assembly is highly fragmented, with gene regions split across scaffolds. This draft assembly in turn limits the depth of the *I. scapularis* genome annotation [62]. If characteristics of the *I. scapularis* draft assembly and annotation, such as gene fragmentation, explain the low homology between *A. americanum* and *I. scapularis*, we predict imperfect matching (<100%) of the *I. scapularis* ESTs to the *I. scapularis* contigs, singletons, and predicted peptides. We tested this prediction by BLAST searches of the 194,460 *I. scapularis* ESTs against these three datasets. They returned between 37% and 66% matching (Table 4). These values are indeed unexpectedly low for intraspecific BLAST searches, supporting fragmentation and low coverage of the *I. scapularis* datasets as a potential source of low homology between *A. americanum* and *I. scapularis*.

Based upon these results for homology between *I. scapularis* datasets (Table 4), a proportion of all *A. americanum* ESTs that failed to match the *I. scapularis* predicted peptides, contigs, and singletons may be discounted as reflecting characteristics of the *I. scapularis* reference rather than low homology. If a proportion of the *A. americanum* ESTs that failed to match the *I. scapularis* datasets is discarded to control for reference quality, higher values for homology between *A. americanum* and *I. scapularis* are estimated: 34% matching between *A. americanum* ESTs and the *I. scapularis* predicted peptides, 35% matching with *I. scapularis* contigs, and 43% matching with *I. scapularis* singletons. We therefore conclude that genetic distance of *A. americanum* and *I. scapularis*, compounded by fragmentation and low coverage of the reference databases of *I. scapularis,* contributes significantly to the high representation of unknown genes in *A. americanum*.

## *Other tick species*

Transcriptomes from additional tick species allowed further investigation of divergence between tick species [24,63-66]. We attempted to investigate if the low homology detected here between *A. americanum* and *I. scapularis* potentially results from divergence between the Metastriata and Prostriata ticks. Four additional BLAST searches were conducted against the EST libraries of *Ixodes ricinus* (castor bean tick) (Prostriata), *Dermacentor variabilis* (American dog tick), *Rhipicephalus microplus*, and *R. appendiculatus* (brown ear tick) (Metastriata). EST libraries were obtained from GenBank, and BLAST searches were conducted individually against each of these four databases. The number of matches with *A. americanum* ESTs was very low: 44 (0.3%) against *I. ricinus*, 52 (0.4%) against *D. variabilis*, 270 (1.9%) against *R. microplus*, and 174 (1.2%) against *R. appendiculatus*. This low percent matching, as compared to *I. scapularis,* is likely due to the small size of the datasets rather than decreased gene conservation (Additional file 1: Table S3). The size of these datasets precludes any further inference of the degree of divergence between Metastriata and Prostriata ticks. More genomic data for tick species are required to accurately conduct comparative studies [62].

## *Tetranychus urticae*

In the course of annotating our *A. americanum* EST dataset, the draft genome sequence of the two-spotted spider mite, *Tetranychus urticae*, was published [6], making it the only chelicerate and *A. americanum*’s closest relative with a published genome. With the *T. urticae* genome, we were able to test gene conservation within the subclass Acari, which contains the ticks and the mites. Two datasets represented the *T. urticae* genome: the predicted peptides and the genome scaffold sequences. BLAST searches of the 14,310 *A. americanum* ESTs returned 2,338 matches (16%) to the predicted peptides and 2,155 (15%) to the main genome (Additional file 3: Figure S2). As in *I. scapularis*, the vast majority of these matches were annotated in UniProtKB: 98.2% for the predicted peptide matches and 97.8% for the main genome matches. This low homology between *Amblyomma* and *Tetranychus* further supports divergence within the Acari as a major source of unknown genes. Indeed, Fukuchi and Nishikawa [13] demonstrate that a lack of close relatives in genomic datasets, as seen with *A. americanum*, is positively correlated with the proportion of unannotated sequences in a dataset.

## What is the biological significance of these unknown genes?

We demonstrate above that when accounting for sequence quality, assembly quality, and gene function, over 50% of the ESTs in this *A. americanum* transcriptome lack homology to genomic datasets of other organisms. The majority of these functional, unknown ESTs lack homology to even the relatively closely-related Acari species *I. scapularis* and *T. urticae*. These unannotated sequences are thus unique to the lineage leading to Metastriata ticks and, perhaps, more specifically to *Amblyomma* species. Further genomic resources must be developed for other Prostriata and Metastriata tick species to evaluate the degree of taxonomic restriction of these orphan genes.

The unique ecology and life-history of tick species suggests ample opportunity for lineage-specific adaptations. For example, the blood-feeding habit is the most prominent characteristic of ticks, having been adopted by only a few other arthropod lineages (e.g. mosquitoes and other dipterans, mites, fleas, lice, bedbugs, some hemipterans) [67]. Genomic data are abundant for many blood-feeders that vector diseases, and these data can be used to evaluate the hypothesis that genes associated with the blood-feeding strategy are lineage-specific. The ARP2 dataset includes gene families from five blood-feeding arthropods: *A. aegypti, A. gambiae, C. quinquefasciatus, I. scapularis*, and the human louse *Pediculus humanus*. Of the 28,769 ARP2 gene families, 4,428 (15%) are exclusive to blood-feeding arthropods. For thirty random combinations of five species from the 14 composing the ARP2 dataset, the average number of exclusive gene families was 4,639.5 (16%). The number and proportion of gene families exclusive to blood-feeders lies well within a single standard deviation of this average and is in fact smaller, indicating that arthropods sharing the blood-feeding habit do not share a greater number of gene families. This suggests that genes associated with blood-feeding may have a high likelihood of being lineage-specific, perhaps due to rapid divergence [10,51] under strong selection exerted by coevolving hosts [68,69]. Additionally, of the 4,428 gene families exclusive to blood-feeding arthropods, most gene families (n = 1,753) are exclusive to a single species. Only one gene family is shared among all five species and only 23 among four. When three species share a gene family (n = 1,148), the species are most commonly the three closely-related mosquitoes.

By BLAST searching against the *I. scapularis* peptides, 173 of these blood-feeding exclusive gene families were identified in the *A. americanum* EST library. This is a small fraction (5%) of the total gene families exclusive to blood-feeding arthropods that were identified in *I. scapularis* (n = 3,554) (Additional file 1: Table S4). This result corroborates the low homology observed between *A. americanum* and *I. scapularis*. Moreover, it supports adaptation to blood-feeding as one potential source of unannotated ESTs in this *A. americanum* transcriptome. We develop the blood-feeding habit here as an example of a trait that defines tick ecology and life-history. A general challenge for ecological and evolutionary genomics is to carry out the necessary functional genetic experiments to evaluate the hypothesis that unannotated sequences are linked to an organism’s unique biology.

## Conclusions

Here, we present a characterized set of ESTs for the hard tick *A. americanum* representing an estimated 14,310 unique sequences. The number of ESTs publicly available for this important North American disease vector is more than doubled by this study. The genomic resources available for *A. americanum* remain limited, however, and our results emphasize the need for an annotated gnome assembly for this species to obtain a more comprehensive representation of its genome. The ESTs we report here will prove a powerful resource in annotation of this future *A. americanum* genome.

Additionally, we reveal up to 5,261 functional genes for which no arthropod or tick homologs are currently available. Using the framework outlined above, only 398 unnanotated ESTs could be definitively eliminated due to poor sequence quality, leaving up to 70% (n = 9,749) of ESTs unannotated. Secondly, our assessment of assembly quality found no evidence for selective amplification of unannotated sequences. Thirdly, we establish that lack of annotation does not arise solely from a lack of gene function. A microarray experiment revealed that 59% (n = 5,252) of functional ESTs are unannotated. Finally, low homology to *I. scapularis* and *T. urticae*, the closest arthropod species with significant genomic resources, showed that taxonomic isolation may contribute significantly to the high representation of unknown genes in the *A. americanum* library. Summarizing across each step of this analysis, our results suggest that the proportion of unannotated, functional genes in this *A. americanum* transcriptome exceeds 50%. These unannotated sequences may thus represent genes unique to the *Amblyomma* or Metastriata tick lineages. As taxonomic isolation is reduced by future genome assemblies for *A. americanum* and other hard ticks, the degree of lineage-specific adaptations within tick taxa must be evaluated more closely.

In conclusion, we present a broad overview of *A. americanum* genomics and contribute EST sequences for the development of tick genomics, functional annotation of tick genomic sequences, and enhancement of biological understanding of these major disease vectors. Our findings further confirm that tick lineages are highly divergent [58,59], necessitating whole-genome sequencing of multiple hard tick species. We commend the i5k Insect and other Arthropod Genome Sequencing Initiative. As of January 2013, 8% of arthropod species nominated for sequencing under this initiative belong to the Chelicerata, the largest representation by any sub-phylum other than the Hexapoda (http://www.arthropodgenomes.org/wiki/i5K). These genomic efforts will both enhance knowledge and facilitate the development of management strategies for tick-borne illnesses. Moreover, we offer a framework for the evaluation of unannotated sequences that can be applied widely, to genomes and transcriptomes of a diversity of taxa. The extension of genomic resources across the tree of life calls for recognition of the significance of unannotated sequences in genomic datasets and for thorough analysis of their biological function.

## Methods

### EST sampling and sequencing

Ticks from five developmental stages (larvae, nymph, adult male, adult female, engorged female) were obtained from the Oklahoma State University Tick Rearing Facility. Additional information regarding these lab-reared colonies can be found at http://www.reeis.usda.gov/web/crisprojectpages/0160810-centralized-tick-rearing.html. A natural population of adult ticks (50 males and 50 females) was collected from a field site in Solsberry, Owen County, Indiana, USA. Collections were made in late spring by dragging on a section of private property characterized by a patchwork of old field and young forest vegetation. This group is referred to as “wild-collected.”

Total RNA from each sample group (adult male, adult female, engorged female, nymph, larval, wild-collected) was isolated using TRIzol reagent (Invitrogen Life Sciences, Carlsbad, CA) and purified using the RNeasy kit (Qiagen, Valencia, CA). Following removal of DNA and other contaminants with DNAfree (Ambion, Life Sciences, Carlsbad, CA), each RNA sample was quantified with a Nanodrop and qualified with an Agilent Bioanalyzer. Double-stranded cDNA was constructed and normalized from these six isolated, purified RNA samples using the Trimmer-Direct Normalization Kit (Evrogen, Moscow, Russia) in conjunction with the Creator SMART cDNA Library Construction Kit (Clontech, Mountain View, CA). Normalized libraries were biased towards larger sequences by fractionating the cDNA with CHROMA-SPIN 400 columns (Creator SMART kit) following Sfil digestion. Normalized and digested cDNA was directionally cloned into pDRN-LIB vectors (Clontech). These cDNA inserts were flanked by the Sfil A (5’-GGCCATTACGGCC-3’) and Sfil B (5’-GGCCGCCTCGGCC-3’) linker sequences. Following ligation, cloned inserts were transformed into TOP10 Electrocomp cells (Invitrogen) via electroporation. Cells were plated onto LB/agar containing 50 μg/mL chloramphenicol and grown overnight at 37°C.

For each library, a 384-well glycerol stock plate was hand-picked for quality assurance testing. A random subset of 864 clones, representing at minimum, 96 samples from each plate/library, were plasmid-prepped with the PerfectPrep Direct Bind Plasmid Kit (Eppendorf). To determine the sizes of these control samples, each selected cDNA insert was PCR amplified and visualized using agarose gel electrophoresis and a Kodak 440cf imaging station. The average molecular weight of this subset was determined to be 1,185 bp. Normalization efficiency was then assessed by sequencing a single pass 5’ read of each cDNA insert using BigDye Terminator ver3.1 sequencing chemistry. Raw trace files were converted using phred2fasta and all reads qualified with TIGR Lucy [70], which trims low quality reads, short reads, vector-only sequences, and mitochondrial reads. Mitochondrial reads were specifically trimmed using the *Amblyomma triguttatum* mitochondrial genome. A total of 36 inserts were dropped due to sequencer failure, and an additional 32 were excluded due to absence of an insert or short sequence (<100 bp). The remaining 796 trimmed reads were assembled with CAP3 [48], yielding 787 contigs and singletons. This initial result corresponds to a gene discovery rate of 99% among libraries.

After quality assurance, colonies from each of the six libraries were arrayed into 384-well glycerol stock plates and sent to the Genomics Core Facility at Purdue University (Additional file 1: Table S1). Plasmid DNA was amplified from plates using the TempliPhi Rolling Circle Amplification Kit (GE Healthcare, Piscataway, NJ) and sequenced on ABI3730 sequencers using BigDye Terminator ver3.1 sequencing chemistry. The primer pDNRlib30-50fwd (5’-TATACGAAGTTATCAGTCGACG-3’) was used for sequencing. The raw trace files were processed with the ESTPiper (http://cas-bioinfo.cas.unt.edu/estpiper/index.html) [47], a web-based analysis tool that processes and assembles ESTs from raw trace files through phred2fasta, TIGR Lucy, CAP3, and smaller trimming programs developed by the Center for Genomics and Bioinformatics, Indiana University.

### Assembly

A total of 20,256 cDNA samples were generated from the six normalized *A. americanum* libraries (Additional file 1: Table S2a). Trace files were subjected to base calling with phred and data cleaning through the ESTPiper analysis tool, resulting in removal of 4,866 low-quality sequences. The ESTPiper removed low quality reads, short reads (< 100 bp), reads without inserts, vector-only sequences, and mitochondrial sequences. PolyA trimming was applied to only 16% of sequences during data cleaning, indicating the presence of continuous polyA sequences greater than 15 bp. The longest polyA sequence trimmed was 150 bp in length. The remaining 15,390 high quality sequences were submitted to the ESTPiper’s CAP3 component for assembly, with overlap match set at 90%. The resulting 12,319 original ESTs were then secondarily assembled, using the ESTPiper’s CAP3 assembler and an overlap match of 90%, with the 6,502 ESTs reported by previous studies available at NCBI’s GenBank [45] (Additional file 1: Table S2b). Sequences from the six normalized libraries are publicly available through GenBank [dbEST: JZ168803-JZ170971, JZ170972-JZ173026, JZ173027-JZ175168, JZ175169-JZ177905, JZ177906-JZ180320, and JZ180321-JZ183760, corresponding to adult female, adult male, engorged female, larval, nymph, and wild-collected transcripts, respectively]. Assembled sequences have been deposited in the Transcriptome Shotgun Assembly database [GenBank TSA: GAGD01000000].

### Annotation

The ESTPiper was used to perform a tBLASTx search of the *A. americanum* EST library against proteins in the UniProtKB database [49] with an e-value threshold of 1x 10^-5^. All returns shorter than 33 amino acids were removed. These quality thresholds were applied for all BLAST searches reported in this study.

A BLAST search of the *A. americanum* EST library was also performed against a combined database of the predicted peptides of nine insect species, *Acyrthosiphon pisum* (pea aphid), *Aedes aegypti* (yellow fever mosquito), *Anopheles gambiae* (African malaria mosquito), *Apis mellifera* (honey bee), *Bombyx mori* (silkmoth), *Culex quinquefasciatus* (Southern house mosquito), *Drosophila melanogaster* (common fruit fly), *Pediculus humanus* (human louse), and *Tribolium castaneum* (red flour beetle), one crustacean, *Daphnia pulex* (water flea), and two chelicerates, *I. scapularis* (black-legged deer tick) and the recently sequenced *Tetranychus urticae* (two-spotted spider mite). These datasets were obtained, respectively, from AphidBase [71], VectorBase (VectorBase [72], http://www.vectorbase.org, *A. aegypti* Liverpool LVP annotation, Aaegl1), VectorBase (*A. gambiae* PEST annotation, AgamP3.5), BeeBase [73], SilkDB [74], VectorBase (*C. quinquefasciatus* Johannesburg annotation, CpipJ1), FlyBase [75], VectorBase (*P. humanus* USDA annotation, PhumU1), Beetlebase [76], wFleaBase [77], VectorBase (*I. scapularis* WIKEL annotation), and BOGAS (http://bioinformatics.psb.ugent.be/webtools/bogas/overview/Tetur, *T. urticae*).

### Framework for investigation of unknown genes

#### Hypothesis 1: sequence quality

ORFs, ORF lengths, and start codons were predicted for ESTs using the OrfPredictor [78] component of the Transcriptome Analysis Pipeline of the Integrative Services for Genomic Analysis (ISGA) at Indiana University’s Center for Genomics and Bioinformatics [79]. Nucleotide lengths and GC-contents were estimated using Geneious [80]. ORF nucleotide lengths, EST nucleotide lengths, and GC-contents of annotated and unannotated ESTs were compared using Welch’s T-tests in R v2.12.2.

#### Hypothesis 2: assembly quality

ARP2 IDs for arthropod gene families and copy numbers were obtained from eugenes/Arthropods (http://arthropods.eugenes.org/arthropods/) [7]. This database reports gene families shared between 14 arthropod species: 12 insect species (*A. pisum, A. aegypti, A. gambiae, A. mellifera, B. mori, C. quinquefasciatus, D. melanogaster, D. pseudoobscura, D. mojavensis*, *Nasonia vitripennis* (jewel wasp)*, P. humanus*, and *T. castaneum*), one crustacean species (*D. pulex*), and one chelicerate species (*I. scapularis*). The inclusion of *I. scapularis* in the ARP2 dataset allowed ARP2 IDs to be assigned to *A. americanum* ESTs. ESTs were first matched to the *I. scapularis* peptide dataset (see Methods section below, “Phylogenetic distance between *A. americanum* and other arthropods”). *Amblyomma americanum* ESTs with a match to *I. scapularis* were then assigned the ARP2 ID associated with the *I. scapularis* match. Single-copy ARP2 gene families were extracted by selecting gene families present in all 14 arthropod species with only one copy. The assembly failure rate was calculated by multiplying 1) the proportion of single-copy genes that were multi-copy in the *A. americanum* EST assembly with 2) the proportion of single-copy genes represented by the set of erroneous multi-copy genes in the assembly.

To determine the contribution of assembly quality to the percentage of unannotated sequences, two alternate assemblies were produced by assembling the unassembled 12,319 ESTs reported by this study with GenBank’s 6,502 *A. americanum* ESTs. The two alternate assemblies were produced using Newbler (454 Life Sciences) and the updated CAP3 assemblers. These assemblies were then annotated as described previously and contrasted with results for annotation of the original assembly.

#### Hypothesis 3: functional expression of A. americanum ESTs

A custom designed microarray was manufactured on the Roche NimbleGen (Madison, WI) multiplex (12-plex) long-oligonucleotide (60 nt) platform. Each glass slide contains 12 identical arrays prepared using a Maskless Array Synthesizer [81]. Each array consists of 137,000 temperature-balanced probes; 13,928 assembled contigs and singletons are represented by nine unique probes, 145 are represented by < 9 unique probes. The array also contains control probes and 11,657 random probes designed to reflect the genome nucleotide composition by Markov modeling to experimentally determine the appropriate thresholds that measure significant hybridization signals over the background.

RNA from replicated adult, nymph and larval stage animals, both with and without *Rickettsia* infection, was extracted in TRIzol® following manufacturer's directions (Invitrogen, Carlsbad, CA). The microarray protocol follows previously published methods [82,83]. Raw microarray data were processed with the limma package [84] in R version 2.9.0 [85] to normalize expression scores.

To determine if a gene was expressed, we calculated the 99.5% quantile for expression score of random probes in each individual as the cutoff for calling expression. Thus, for each sample, a called expression is significant at a p-value of 0.005. For each contig and singleton with more than one probe, we tested the median probe value against this threshold. With this, we determined whether or not a gene had expression support in any of the treatments.

#### Hypothesis 4: phylogenetic distance between A. americanum and other arthropods

To determine homology between *A. americanum* and *I. scapularis*, several BLAST searches of the *A. americanum* EST library were conducted against various datasets of the *I. scapularis* genome sequence, which is publicly available through VectorBase. The *I. scapularis* whole-genome sequence assembly and annotation projects are a joint effort of the Broad Institute and the J. Craig Venter Institute. Vectorbase supplies *I. scapularis* genome sequences (570,637 contigs and 7,002,324 unassembled singletons, ~4× coverage of genome), a library of 194,460 ESTs, and 20,486 predicted peptides, which are a combination of sequences from the *I. scapularis* EST library and predicted peptides from the available genome sequences. The *A. americanum* EST library was separately BLASTed against each of these four datasets – contigs, singletons, ESTs, and predicted peptides. These results were then compared to the *A. americanum* BLAST search against the UniProtKB protein database to assess the proportion of *I. scapularis* matches with UniProtKB annotation.

Additionally, BLAST searches of the *I. scapularis* ESTs against *I. scapularis* genomic datasets (contigs, singletons, and predicted peptides) were conducted to correct for the qualityof the *I. scapularis* genome assembly. The number of *A. americanum* ESTs that failed to match against an *I. scapularis* dataset was then multiplied by the proportion of *I. scapularis* ESTs matching that same dataset, in order to discount the proportion of ESTs that may have failed to match due to fragmentation or low coverage of the *I. scapularis* genome assembly. After removing these non-matches, the percent matching of the *A. americanum* EST library against the *I. scapularis* datasets was re-calculated.

Four additional BLAST searches were conducted against EST datasets for one Prostriata (*I. ricinus*) and three Metastriata (*D. variabilis*, *R. microplus,* and *R. appendiculatus*) ticks. All EST datasets were obtained from GenBank,

Finally, BLAST searches of the *A. americanum* EST library were also conducted against two datasets of the *T. urticae* genome. Genomic data was produced at the Department of Energy Joint Genome Institute (Walnut Creek, CA, USA). The main genome (640 scaffolds, 89.6 megabases, ~8× coverage) and the predicted peptides (18,414) of *T. urticae* are available at (http://bioinformatics.psb.ugent.be/webtools/bogas/overview/Tetur) [6]. Separate BLAST searches of the *A. americanum* EST library were conducted against the main genome and the predicted peptide datasets as described for the *I. scapularis* datasets.

### Biological significance of unknown genes: blood-feeding

eugenes/Arthropods reports gene families for five species of blood-feeding arthropods: *A. aegypti, A. gambiae, C. quinquefasciatus, P. humanus, and I. scapularis*. Gene families found in any of these species and none of the nine non-blood-feeding arthropod taxa were classified as “exclusive” to blood-feeding taxa. To determine if blood-feeding taxa share a disproportionate number of gene families, the number of gene families exclusive to the five blood-feeding taxa was compared to the average number of gene families shared by 30 randomly-generated combinations of five of the 14 species included in eugenes/Arthropods. Gene families exclusive to these five blood-feeding arthropods were identified in *A. americanum* using a BLAST search against the *I. scapularis* peptides, as described under Hypotheses 2 and 4.

## Competing interests

The authors declare that they have no competing interests.

## Authors’ contributions

AKG: Assembled and annotated EST library. Performed data analysis, including summary of sequence and assembly quality, comparisons of datasets, and statistical analyses. Participated in design and coordination of study. Drafted the manuscript. ZS: Created the EST library. Contributed to assembly, annotation, and summary of sequence quality. Participated in design and coordination of study. Helped to draft the manuscript. CF: Conceived of the study, participated in its design and coordination, and helped to draft the manuscript. KC: Conceived of the study, participated in its design and coordination, and helped to draft the manuscript. JKC: Participated in assembly, annotation, and data analysis. Performed microarray construction and analysis. Conceived of the study, participated in its design and coordination, and helped to draft the manuscript. All authors read and approved the final manuscript.

## Supplementary Material

Additional file 1 **Tables.** Includes tables associated with the main text and the Supplemental Text Click here for file

Additional file 2 **Supplemental Text.** Includes discussions of the *A. americanum* EST dataset deconstructed according to life stage, as well as additional annotation results. Click here for file

Additional file 3 **Figures.** Figures S1 through S4 and associated legends. Click here for file
