# Supplementary material for: Why so many unknown genes? Partitioning orphans from a representative transcriptome of the lone star tick Amblyomma americanum
Source: BMC Genomics. 2013 Feb 27;14:135. doi: 10.1186/1471-2164-14-135 (PMC3616916; doi:10.1186/1471-2164-14-135)
Supplement: Additional file 1 — Tables. Includes tables associated with the main text and the Supplemental Text [file 1471-2164-14-135-S1.docx]

SUPPLEMENTAL TABLES

| **Supplemental Table 1: Number of clones sequenced for each individual tick library.** cDNA libraries were constructed from five developmental stages reared under laboratory conditions. An additional library was constructed from wild-collected adult males (N=50) and adult females (N=50) |
| --- |
| \| **Library** \| **No. clones** \| \| --- \| --- \| \| Larvae \| 3,072 \| \| Nymph \| 3,072 \| \| Adult Male \| 3,072 \| \| Adult Female \| 3,072 \| \| Engorged Female \| 2,976 \| \| Wild-collected \| 4,992 \| |

| **Supplemental Table 2: Summary of the *Amblyomma americanum* EST library primary and secondary assemblies.** Statistics are provided for (A) the primary assembly of the *A. americanum* EST library created at the Center for Genomics and Bioinformatics (CGB) and (B) the secondary assembly of the CGB assembly with 6,502 *A. americanum* ESTs available on NCBI’s GenBank. |
| --- |
| \|  \|  \| **Number** \| \| --- \| --- \| --- \| \| 1. **1° Assembly: CGB** \| High quality reads \| 20,256 \| \| Empty sequences discarded \| 3,898 \| \| Short/no sequences discarded \| 45 \| \| Vector-only sequences discarded \| 639 \| \| multiA/T sequences discarded \| 39 \| \| Mitochondrial sequences discarded \| 208 \| \| *Escherichia coli* sequences discarded \| 21 \| \| Univec filter sequences discarded \| 0 \| \| Short sequences (< 100 bp) discarded \| 15 \| \| Sequences input into CAP3 \| 15,390 \| \| Average nucleotide length of output sequences \| 557 \| \| Total bases covered by 1° assembly \| 6,861, 683 \| \| Singletons following 1° assembly \| 10,443 \| \| Average nucleotide length of singletons \| 521.8 \| \| Contigs following 1° assembly \| 1,876 \| \| Average nucleotide length of contigs \| 752.8 \| \| **1° assembly: total unique sequences** \| **12,319** \| \| 1. **2° Assembly:**   **CGB +** **GenBank** \| GenBank ESTs prior to 2° assembly \| 6,502 \| \| Total ESTs prior to 2° assembly \| 18,821 \| \| Average nucleotide length of output sequences \| 553 \| \| Total bases covered by 2° assembly \| 7,913,430 \| \| Singletons following 2° assembly \| 11,580 \| \| Average nucleotides length of singletons \| 508.7 \| \| Contigs following 2° assembly \| 2,730 \| \| Average nucleotide length of contigs \| 740.7 \| \| **2° assembly: total unique sequences** \| **14,310** \| |

| **Supplemental Table 3: Summary of BLAST searches of *Amblyomma americanum* ESTs against other tick species.** EST databases for each species were obtained through NCBI GenBank. Reported here are the number of ESTs in each species’ dataset, the number of matches obtained through BLAST searches against the *A. americanum* EST library, and the proportion of the *A. americanum* library with a match in parentheses. |
| --- |
| \| **Tick species** \| **Number ESTs** \| **Match against *A. americanum*** \| \| --- \| --- \| --- \| \| *Ixodes scapularis* \| 194,460 \| 3,592 (25.1%) \| \| *Ixodes ricinus* \| 2,044 \| 44 (0.3%) \| \| *Dermacentor variabilis* \| 2,150 \| 52 (0.4%) \| \| *Rhipicephalus microplus* \| 52,902 \| 270 (1.9%) \| \| *Rhipicephalus appendiculatus* \| 19,185 \| 172 (1.2%) \| |

| **Supplemental Table 4: ARP2 gene families exclusive to *A. americanum, I. scapularis*, and four blood-feeding insects.** Columns of numbers indicate the number of genes per family per species. “# Taxa” indicates the number of blood-feeding taxa, excluding *A. americanum*, in which the gene family is present. ARP2 IDs are ranked in descending order according to the number of taxa represented. Abbreviations correspond to blood-feeding taxa: *Aam (A. americanum)* from the EST library*; Isc (I. scapularis); Aae (A. aegypti); Aga (A. gambiae); Cqu (C. quinquefasciatus); Phu (P. humanus)* from eugenes/Arthropods*.* Annotations obtained from eugenes/Arthropods. |
| --- |
| \| **GeneID** \| ***Aam*** \| ***Isc*** \| ***Aae*** \| ***Aga*** \| ***Cqu*** \| ***Phu*** \| **# Taxa** \| **Annotation** \| \| --- \| --- \| --- \| --- \| --- \| --- \| --- \| --- \| --- \| \| ARP2_G15441 \| 1 \| 1 \| 0 \| 1 \| 1 \| 0 \| 3 \| hypothetical protein \| \| ARP2_G9988 \| 1 \| 1 \| 1 \| 0 \| 2 \| 0 \| 3 \| hypothetical protein; tamo \| \| ARP2_G14745 \| 1 \| 1 \| 1 \| 0 \| 1 \| 0 \| 3 \| transcription initiation factor tfiid subunit; transcription initiation factor tfii-d subunit \| \| ARP2_G21320 \| 1 \| 1 \| 0 \| 1 \| 0 \| 0 \| 2 \| 40s ribosomal protein s11 \| \| ARP2_G21216 \| 1 \| 1 \| 0 \| 1 \| 0 \| 0 \| 2 \| carnitine acyltransferase \| \| ARP2_G27667 \| 1 \| 1 \| 0 \| 0 \| 0 \| 1 \| 2 \| gtp-binding nuclear protein ran1 \| \| ARP2_G27867 \| 1 \| 1 \| 0 \| 0 \| 0 \| 1 \| 2 \| hepatoma up-regulated protein \| \| ARP2_G24348 \| 1 \| 1 \| 0 \| 0 \| 1 \| 0 \| 2 \| hypothetical protein \| \| ARP2_G20913 \| 1 \| 1 \| 1 \| 0 \| 0 \| 0 \| 2 \| lipase maturation factor \| \| ARP2_G27774 \| 1 \| 1 \| 0 \| 0 \| 0 \| 1 \| 2 \| microphthalmia-associated transcription factor \| \| ARP2_G21306 \| 1 \| 1 \| 0 \| 1 \| 0 \| 0 \| 2 \| neutral sphingomyelinase n-smase activation associated factor fan \| \| ARP2_G24508 \| 1 \| 1 \| 0 \| 0 \| 1 \| 0 \| 2 \| nuclear receptor \| \| ARP2_G24378 \| 1 \| 1 \| 0 \| 0 \| 1 \| 0 \| 2 \| serine/threonine protein phosphatase 4 regulatory subunit \| \| ARP2_G24204 \| 2 \| 1 \| 0 \| 0 \| 1 \| 0 \| 2 \| stretchin-mlckcg18255; myomesin \| \| ARP2_G27487 \| 1 \| 1 \| 0 \| 0 \| 0 \| 1 \| 2 \| zinc finger protein \| \| ARP2_G27824 \| 1 \| 1 \| 0 \| 0 \| 0 \| 1 \| 2 \| zinc finger protein fyve domain containing protein \| \| ARP2_G27625 \| 1 \| 2 \| 0 \| 0 \| 0 \| 0 \| 1 \| 15-hydroxyprostaglandin dehydrogenase \| \| ARP2_G313 \| 1 \| 34 \| 0 \| 0 \| 0 \| 0 \| 1 \| 4-coumarate-coa ligase; amp dependent coa ligase; amp dependent ligase \| \| ARP2_G11895 \| 1 \| 5 \| 0 \| 0 \| 0 \| 0 \| 1 \| abc transporter \| \| ARP2_G5987 \| 5 \| 13 \| 0 \| 0 \| 0 \| 0 \| 1 \| acetylcholinesterase; esterase; jhedup \| \| ARP2_G10603 \| 1 \| 6 \| 0 \| 0 \| 0 \| 0 \| 1 \| amp dependent coa ligase \| \| ARP2_G27652 \| 1 \| 2 \| 0 \| 0 \| 0 \| 0 \| 1 \| antigen b membrane protein \| \| ARP2_G27538 \| 1 \| 2 \| 0 \| 0 \| 0 \| 0 \| 1 \| beta-1,4-n-acetylgalactosaminyl transferase bre-4 \| \| ARP2_G19557 \| 2 \| 3 \| 0 \| 0 \| 0 \| 0 \| 1 \| carrier protein \| \| ARP2_G27807 \| 3 \| 2 \| 0 \| 0 \| 0 \| 0 \| 1 \| centromere protein b \| \| ARP2_G14100 \| 3 \| 4 \| 0 \| 0 \| 0 \| 0 \| 1 \| chitinase \| \| ARP2_G19480 \| 1 \| 3 \| 0 \| 0 \| 0 \| 0 \| 1 \| class b secretin-like g-protein coupled receptor gprmth4; class b secretin-like g-protein coupled receptor gprmth1; class b secretin-like g-protein coupled receptor gprmth3 \| \| ARP2_G19482 \| 2 \| 3 \| 0 \| 0 \| 0 \| 0 \| 1 \| coagulation factor precursor \| \| ARP2_G4941 \| 7 \| 14 \| 0 \| 0 \| 0 \| 0 \| 1 \| cystatin \| \| ARP2_G1446 \| 6 \| 18 \| 0 \| 0 \| 0 \| 0 \| 1 \| cytochrome p450 \| \| ARP2_G5984 \| 2 \| 13 \| 0 \| 0 \| 0 \| 0 \| 1 \| cytochrome p450 \| \| ARP2_G5988 \| 1 \| 13 \| 0 \| 0 \| 0 \| 0 \| 1 \| cytochrome p450 \| \| ARP2_G7798 \| 2 \| 10 \| 0 \| 0 \| 0 \| 0 \| 1 \| cytochrome p450 \| \| ARP2_G9665 \| 2 \| 7 \| 0 \| 0 \| 0 \| 0 \| 1 \| cytochrome p450 \| \| ARP2_G11912 \| 4 \| 5 \| 0 \| 0 \| 0 \| 0 \| 1 \| cytochrome p450 \| \| ARP2_G19442 \| 4 \| 3 \| 0 \| 0 \| 0 \| 0 \| 1 \| cytochrome p450 \| \| ARP2_G19463 \| 1 \| 3 \| 0 \| 0 \| 0 \| 0 \| 1 \| cytochrome p450 \| \| ARP2_G27689 \| 1 \| 2 \| 0 \| 0 \| 0 \| 0 \| 1 \| cytochrome p-450 \| \| ARP2_G19484 \| 1 \| 3 \| 0 \| 0 \| 0 \| 0 \| 1 \| dehydrogenase \| \| ARP2_G27850 \| 1 \| 2 \| 0 \| 0 \| 0 \| 0 \| 1 \| down syndrome cell adhesion molecule; sdk; contactin-like cell adhesion molecule \| \| ARP2_G14081 \| 1 \| 4 \| 0 \| 0 \| 0 \| 0 \| 1 \| endothelial-specific receptor tyrosine kinase; cell adhesion molecule \| \| ARP2_G11902 \| 2 \| 5 \| 0 \| 0 \| 0 \| 0 \| 1 \| f-box/leucine rich repeat protein \| \| ARP2_G27773 \| 1 \| 2 \| 0 \| 0 \| 0 \| 0 \| 1 \| flavonol reductase/cinnamoyl-coa reductase \| \| ARP2_G27520 \| 1 \| 2 \| 0 \| 0 \| 0 \| 0 \| 1 \| gchfr \| \| ARP2_G27630 \| 1 \| 2 \| 0 \| 0 \| 0 \| 0 \| 1 \| glutathione s-transferase \| \| ARP2_G8936 \| 4 \| 8 \| 0 \| 0 \| 0 \| 0 \| 1 \| heat shock protein \| \| ARP2_G11892 \| 8 \| 5 \| 0 \| 0 \| 0 \| 0 \| 1 \| hebreain \| \| ARP2_G430 \| 7 \| 29 \| 0 \| 0 \| 0 \| 0 \| 1 \| hypothetical protein \| \| ARP2_G1177 \| 3 \| 19 \| 0 \| 0 \| 0 \| 0 \| 1 \| hypothetical protein \| \| ARP2_G2472 \| 1 \| 16 \| 0 \| 0 \| 0 \| 0 \| 1 \| hypothetical protein \| \| ARP2_G7315 \| 1 \| 11 \| 0 \| 0 \| 0 \| 0 \| 1 \| hypothetical protein \| \| ARP2_G9671 \| 1 \| 7 \| 0 \| 0 \| 0 \| 0 \| 1 \| hypothetical protein \| \| ARP2_G11898 \| 2 \| 5 \| 0 \| 0 \| 0 \| 0 \| 1 \| hypothetical protein \| \| ARP2_G14058 \| 1 \| 4 \| 0 \| 0 \| 0 \| 0 \| 1 \| hypothetical protein \| \| ARP2_G14059 \| 4 \| 4 \| 0 \| 0 \| 0 \| 0 \| 1 \| hypothetical protein \| \| ARP2_G14062 \| 1 \| 4 \| 0 \| 0 \| 0 \| 0 \| 1 \| hypothetical protein \| \| ARP2_G14073 \| 1 \| 4 \| 0 \| 0 \| 0 \| 0 \| 1 \| hypothetical protein \| \| ARP2_G14097 \| 1 \| 4 \| 0 \| 0 \| 0 \| 0 \| 1 \| hypothetical protein \| \| ARP2_G19437 \| 6 \| 3 \| 0 \| 0 \| 0 \| 0 \| 1 \| hypothetical protein \| \| ARP2_G19465 \| 1 \| 3 \| 0 \| 0 \| 0 \| 0 \| 1 \| hypothetical protein \| \| ARP2_G19468 \| 4 \| 3 \| 0 \| 0 \| 0 \| 0 \| 1 \| hypothetical protein \| \| ARP2_G19498 \| 1 \| 3 \| 0 \| 0 \| 0 \| 0 \| 1 \| hypothetical protein \| \| ARP2_G19499 \| 1 \| 3 \| 0 \| 0 \| 0 \| 0 \| 1 \| hypothetical protein \| \| ARP2_G19509 \| 1 \| 3 \| 0 \| 0 \| 0 \| 0 \| 1 \| hypothetical protein \| \| ARP2_G19516 \| 1 \| 3 \| 0 \| 0 \| 0 \| 0 \| 1 \| hypothetical protein \| \| ARP2_G19544 \| 1 \| 3 \| 0 \| 0 \| 0 \| 0 \| 1 \| hypothetical protein \| \| ARP2_G19552 \| 1 \| 3 \| 0 \| 0 \| 0 \| 0 \| 1 \| hypothetical protein \| \| ARP2_G19564 \| 2 \| 3 \| 0 \| 0 \| 0 \| 0 \| 1 \| hypothetical protein \| \| ARP2_G19570 \| 2 \| 3 \| 0 \| 0 \| 0 \| 0 \| 1 \| hypothetical protein \| \| ARP2_G27506 \| 1 \| 2 \| 0 \| 0 \| 0 \| 0 \| 1 \| hypothetical protein \| \| ARP2_G27509 \| 1 \| 2 \| 0 \| 0 \| 0 \| 0 \| 1 \| hypothetical protein \| \| ARP2_G27515 \| 2 \| 2 \| 0 \| 0 \| 0 \| 0 \| 1 \| hypothetical protein \| \| ARP2_G27537 \| 1 \| 2 \| 0 \| 0 \| 0 \| 0 \| 1 \| hypothetical protein \| \| ARP2_G27577 \| 1 \| 2 \| 0 \| 0 \| 0 \| 0 \| 1 \| hypothetical protein \| \| ARP2_G27616 \| 2 \| 2 \| 0 \| 0 \| 0 \| 0 \| 1 \| hypothetical protein \| \| ARP2_G27626 \| 1 \| 2 \| 0 \| 0 \| 0 \| 0 \| 1 \| hypothetical protein \| \| ARP2_G27653 \| 1 \| 2 \| 0 \| 0 \| 0 \| 0 \| 1 \| hypothetical protein \| \| ARP2_G27654 \| 1 \| 2 \| 0 \| 0 \| 0 \| 0 \| 1 \| hypothetical protein \| \| ARP2_G27662 \| 1 \| 2 \| 0 \| 0 \| 0 \| 0 \| 1 \| hypothetical protein \| \| ARP2_G27676 \| 1 \| 2 \| 0 \| 0 \| 0 \| 0 \| 1 \| hypothetical protein \| \| ARP2_G27684 \| 1 \| 2 \| 0 \| 0 \| 0 \| 0 \| 1 \| hypothetical protein \| \| ARP2_G27715 \| 2 \| 2 \| 0 \| 0 \| 0 \| 0 \| 1 \| hypothetical protein \| \| ARP2_G27726 \| 1 \| 2 \| 0 \| 0 \| 0 \| 0 \| 1 \| hypothetical protein \| \| ARP2_G27755 \| 1 \| 2 \| 0 \| 0 \| 0 \| 0 \| 1 \| hypothetical protein \| \| ARP2_G27785 \| 6 \| 2 \| 0 \| 0 \| 0 \| 0 \| 1 \| hypothetical protein \| \| ARP2_G27804 \| 5 \| 2 \| 0 \| 0 \| 0 \| 0 \| 1 \| hypothetical protein \| \| ARP2_G27805 \| 1 \| 2 \| 0 \| 0 \| 0 \| 0 \| 1 \| hypothetical protein \| \| ARP2_G27817 \| 1 \| 2 \| 0 \| 0 \| 0 \| 0 \| 1 \| hypothetical protein \| \| ARP2_G27820 \| 3 \| 2 \| 0 \| 0 \| 0 \| 0 \| 1 \| hypothetical protein \| \| ARP2_G27837 \| 3 \| 2 \| 0 \| 0 \| 0 \| 0 \| 1 \| hypothetical protein \| \| ARP2_G27840 \| 1 \| 2 \| 0 \| 0 \| 0 \| 0 \| 1 \| hypothetical protein \| \| ARP2_G27876 \| 1 \| 2 \| 0 \| 0 \| 0 \| 0 \| 1 \| hypothetical protein \| \| ARP2_G27899 \| 1 \| 2 \| 0 \| 0 \| 0 \| 0 \| 1 \| hypothetical protein \| \| ARP2_G6751 \| 2 \| 12 \| 0 \| 0 \| 0 \| 0 \| 1 \| hypothetical protein; 5' nucleotidase \| \| ARP2_G10595 \| 6 \| 6 \| 0 \| 0 \| 0 \| 0 \| 1 \| hypothetical protein; btb/poz domain-containing protein; mgc154338 protein \| \| ARP2_G47 \| 1 \| 93 \| 0 \| 0 \| 0 \| 0 \| 1 \| hypothetical protein; cuticle protein; cpr50cb \| \| ARP2_G768 \| 1 \| 22 \| 0 \| 0 \| 0 \| 0 \| 1 \| hypothetical protein; cuticle protein; cpr50cb \| \| ARP2_G182 \| 3 \| 47 \| 0 \| 0 \| 0 \| 0 \| 1 \| hypothetical protein; cytochrome p450 cyp15a1; cyp304a1 \| \| ARP2_G582 \| 1 \| 25 \| 0 \| 0 \| 0 \| 0 \| 1 \| hypothetical protein; neprilysin \| \| ARP2_G7323 \| 1 \| 11 \| 0 \| 0 \| 0 \| 0 \| 1 \| hypothetical protein; neprilysin \| \| ARP2_G9670 \| 5 \| 7 \| 0 \| 0 \| 0 \| 0 \| 1 \| hypothetical protein; phosphatidylinositol transfer protein sec14; cral/trio domain-containing protein \| \| ARP2_G10601 \| 9 \| 6 \| 0 \| 0 \| 0 \| 0 \| 1 \| hypothetical protein; phosphatidylinositol transfer protein sec14; cral/trio domain-containing protein \| \| ARP2_G1448 \| 5 \| 18 \| 0 \| 0 \| 0 \| 0 \| 1 \| hypothetical protein; pogo family transposase \| \| ARP2_G27748 \| 6 \| 2 \| 0 \| 0 \| 0 \| 0 \| 1 \| hypothetical protein; pogo family transposase \| \| ARP2_G9667 \| 1 \| 7 \| 0 \| 0 \| 0 \| 0 \| 1 \| hypothetical protein; salivary gland metalloprotease \| \| ARP2_G10610 \| 1 \| 6 \| 0 \| 0 \| 0 \| 0 \| 1 \| hypothetical protein; serine protease inhibitor; acp24a4 \| \| ARP2_G2473 \| 11 \| 16 \| 0 \| 0 \| 0 \| 0 \| 1 \| hypothetical protein; traf6; secreted protein \| \| ARP2_G11909 \| 5 \| 5 \| 0 \| 0 \| 0 \| 0 \| 1 \| hypothetical protein; vitellogenin-1 precursor; hemelipoglycoprotein precursor \| \| ARP2_G19466 \| 4 \| 3 \| 0 \| 0 \| 0 \| 0 \| 1 \| hypothetical protein \| \| ARP2_G27609 \| 1 \| 2 \| 0 \| 0 \| 0 \| 0 \| 1 \| hypothetical protein \| \| ARP2_G7322 \| 1 \| 11 \| 0 \| 0 \| 0 \| 0 \| 1 \| hypothetical protein \| \| ARP2_G19515 \| 2 \| 3 \| 0 \| 0 \| 0 \| 0 \| 1 \| hypothetical protein \| \| ARP2_G27742 \| 1 \| 2 \| 0 \| 0 \| 0 \| 0 \| 1 \| hypothetical protein \| \| ARP2_G27782 \| 1 \| 2 \| 0 \| 0 \| 0 \| 0 \| 1 \| hypothetical protein \| \| ARP2_G27790 \| 2 \| 2 \| 0 \| 0 \| 0 \| 0 \| 1 \| hypothetical protein \| \| ARP2_G8946 \| 5 \| 8 \| 0 \| 0 \| 0 \| 0 \| 1 \| immunoglobulin-binding protein \| \| ARP2_G19489 \| 1 \| 3 \| 0 \| 0 \| 0 \| 0 \| 1 \| leucine rich repeat and sterile alpha motif containing 1; hypothetical protein \| \| ARP2_G27610 \| 1 \| 2 \| 0 \| 0 \| 0 \| 0 \| 1 \| limkain b1 \| \| ARP2_G9662 \| 2 \| 7 \| 0 \| 0 \| 0 \| 0 \| 1 \| lipid storage droplets surface-binding protein; hypothetical protein; lipid storage droplets surface binding protein 2 lsd2 \| \| ARP2_G27888 \| 1 \| 2 \| 0 \| 0 \| 0 \| 0 \| 1 \| lipoprotein \| \| ARP2_G14080 \| 1 \| 4 \| 0 \| 0 \| 0 \| 0 \| 1 \| mite group 2 allergen tyr p \| \| ARP2_G55 \| 6 \| 91 \| 0 \| 0 \| 0 \| 0 \| 1 \| monocarboxylate transporter; out; monocarboxylate transporter 1 \| \| ARP2_G1176 \| 9 \| 19 \| 0 \| 0 \| 0 \| 0 \| 1 \| nymphal histamine binding protein b \| \| ARP2_G19469 \| 1 \| 3 \| 0 \| 0 \| 0 \| 0 \| 1 \| paramyosin \| \| ARP2_G27575 \| 1 \| 2 \| 0 \| 0 \| 0 \| 0 \| 1 \| phosphatidylethanolamine binding protein \| \| ARP2_G27633 \| 4 \| 2 \| 0 \| 0 \| 0 \| 0 \| 1 \| polyprotein of retroviral origin \| \| ARP2_G19490 \| 3 \| 3 \| 0 \| 0 \| 0 \| 0 \| 1 \| polyprotein of retroviral origin \| \| ARP2_G27567 \| 1 \| 2 \| 0 \| 0 \| 0 \| 0 \| 1 \| reductase \| \| ARP2_G19476 \| 1 \| 3 \| 0 \| 0 \| 0 \| 0 \| 1 \| reverse transcriptase \| \| ARP2_G27695 \| 2 \| 2 \| 0 \| 0 \| 0 \| 0 \| 1 \| reverse transcriptase \| \| ARP2_G10606 \| 1 \| 6 \| 0 \| 0 \| 0 \| 0 \| 1 \| rna-dependent rna polymerase \| \| ARP2_G14067 \| 7 \| 4 \| 0 \| 0 \| 0 \| 0 \| 1 \| rnase h \| \| ARP2_G8944 \| 2 \| 8 \| 0 \| 0 \| 0 \| 0 \| 1 \| scapularisin preproprotein \| \| ARP2_G7797 \| 8 \| 10 \| 0 \| 0 \| 0 \| 0 \| 1 \| secreted protein \| \| ARP2_G7800 \| 3 \| 10 \| 0 \| 0 \| 0 \| 0 \| 1 \| secreted protein \| \| ARP2_G8373 \| 3 \| 9 \| 0 \| 0 \| 0 \| 0 \| 1 \| secreted protein \| \| ARP2_G8941 \| 4 \| 8 \| 0 \| 0 \| 0 \| 0 \| 1 \| secreted protein \| \| ARP2_G9673 \| 3 \| 7 \| 0 \| 0 \| 0 \| 0 \| 1 \| secreted protein \| \| ARP2_G19445 \| 12 \| 3 \| 0 \| 0 \| 0 \| 0 \| 1 \| secreted protein \| \| ARP2_G19488 \| 4 \| 3 \| 0 \| 0 \| 0 \| 0 \| 1 \| secreted protein \| \| ARP2_G9660 \| 1 \| 7 \| 0 \| 0 \| 0 \| 0 \| 1 \| secreted protein; hypothetical protein \| \| ARP2_G9675 \| 5 \| 7 \| 0 \| 0 \| 0 \| 0 \| 1 \| secreted protein; hypothetical protein \| \| ARP2_G8943 \| 1 \| 8 \| 0 \| 0 \| 0 \| 0 \| 1 \| secreted protein; phospholipase b-like 2 precursor lamina ancestor homolog 2 lama-like protein 2 kda protein p76 kda protein \| \| ARP2_G4943 \| 6 \| 14 \| 0 \| 0 \| 0 \| 0 \| 1 \| secreted salivary gland peptide \| \| ARP2_G5991 \| 5 \| 13 \| 0 \| 0 \| 0 \| 0 \| 1 \| secreted salivary gland peptide \| \| ARP2_G14047 \| 1 \| 4 \| 0 \| 0 \| 0 \| 0 \| 1 \| secreted salivary gland peptide \| \| ARP2_G27693 \| 1 \| 2 \| 0 \| 0 \| 0 \| 0 \| 1 \| secreted salivary gland peptide \| \| ARP2_G4940 \| 5 \| 14 \| 0 \| 0 \| 0 \| 0 \| 1 \| secreted salivary gland peptide; secreted protein \| \| ARP2_G8367 \| 2 \| 9 \| 0 \| 0 \| 0 \| 0 \| 1 \| secreted salivary gland peptide; secreted protein \| \| ARP2_G14077 \| 3 \| 4 \| 0 \| 0 \| 0 \| 0 \| 1 \| secreted salivary gland peptide; secreted protein \| \| ARP2_G14094 \| 2 \| 4 \| 0 \| 0 \| 0 \| 0 \| 1 \| secreted salivary gland peptide; secreted protein \| \| ARP2_G27666 \| 4 \| 2 \| 0 \| 0 \| 0 \| 0 \| 1 \| secreted salivary gland peptide; secreted protein \| \| ARP2_G27794 \| 1 \| 2 \| 0 \| 0 \| 0 \| 0 \| 1 \| sentrin/sumo-specific protease; sentrin/sumo-specific protease senp7 \| \| ARP2_G10599 \| 5 \| 6 \| 0 \| 0 \| 0 \| 0 \| 1 \| serine carboxypeptidase \| \| ARP2_G5985 \| 4 \| 13 \| 0 \| 0 \| 0 \| 0 \| 1 \| serine protease \| \| ARP2_G14103 \| 2 \| 4 \| 0 \| 0 \| 0 \| 0 \| 1 \| serine protease inhibitor serpin; serine protease inhibitor leukocyte elastase inhibitor \| \| ARP2_G19548 \| 1 \| 3 \| 0 \| 0 \| 0 \| 0 \| 1 \| serine protease inhibitor serpin; serine protease inhibitor leukocyte elastase inhibitor \| \| ARP2_G19450 \| 1 \| 3 \| 0 \| 0 \| 0 \| 0 \| 1 \| short chain alcohol dehydrogenase; short-chain alcohol dehydrogenase beta-ketoacyl-acp reductase \| \| ARP2_G27878 \| 1 \| 2 \| 0 \| 0 \| 0 \| 0 \| 1 \| sialin \| \| ARP2_G27704 \| 1 \| 2 \| 0 \| 0 \| 0 \| 0 \| 1 \| slc22a7 protein \| \| ARP2_G7803 \| 1 \| 10 \| 0 \| 0 \| 0 \| 0 \| 1 \| sodium-dependent multivitamin transporter; sodium/solute symporter \| \| ARP2_G11897 \| 1 \| 5 \| 0 \| 0 \| 0 \| 0 \| 1 \| sugar transporter \| \| ARP2_G27650 \| 1 \| 2 \| 0 \| 0 \| 0 \| 0 \| 1 \| sulfonylurea receptor \| \| ARP2_G27511 \| 1 \| 2 \| 0 \| 0 \| 0 \| 0 \| 1 \| ubiquitin-conjugating enzyme \| \| ARP2_G11911 \| 3 \| 5 \| 0 \| 0 \| 0 \| 0 \| 1 \| vesicular amine transporter \| \| ARP2_G14055 \| 1 \| 4 \| 0 \| 0 \| 0 \| 0 \| 1 \| y box binding protein \| \| ARP2_G7316 \| 3 \| 11 \| 0 \| 0 \| 0 \| 0 \| 1 \| zinc finger protein \| \| ARP2_G19436 \| 1 \| 3 \| 0 \| 0 \| 0 \| 0 \| 1 \| zinc finger protein \| \| ARP2_G19527 \| 2 \| 3 \| 0 \| 0 \| 0 \| 0 \| 1 \| zinc finger protein \| \| ARP2_G14095 \| 1 \| 4 \| 0 \| 0 \| 0 \| 0 \| 1 \| zinc finger protein; mcg121035; kr-h1 \| |
|  |
|  |
|  |
|  |

**Supplemental Table 5: Identification of 31 contigs significantly enriched in sequences from individual libraries of *Amblyomma americanum*.** Annotation, enrichment score, ARP2 ID, and description are provided as available. Contigs enriched in sequences from individual libraries were detected by significant deviation from the expectation of equal distribution of a contig’s sequences across libraries. The dominant library(ies) in an enriched contig were those from which 35% or more of sequences were derived. The six libraries are nymph, larvae, adult male, adult female, engorged female, and wild-collected ticks. Annotations obtained from UniProtKB and reference arthropod datasets (see Table 1b).

| **Library** | **Enrichment (χ2)** | **ARP2_ID** | **Protein Description** |  |
| --- | --- | --- | --- | --- |
| Engorged female | 168.5 | G4129 | Programmed cell-death protein |  |
| Wild-collected/adult male | 83.9 |  | Glean peptide 35709 |  |
| Wild-collected | 41.0 | G1548 | 60s acidic ribosomal protein p1 |  |
| Larvae | 37.6 | G3854 | Translationally-controlled tumor protein homolog tctp; histamine release protein |  |
| Wild-collected | 31.0 | G1673 | 60s acidic ribosomal protein l18 |  |
| Wild-collected | 30.8 |  | Glycine proline-rich secreted protein |  |
| Wild-collected | 30.8 | G1523 | 60s ribosomal protein l10 |  |
| Wild-collected/adult male | 30.2 |  | Glean peptide 20330 |  |
| Wild-collected | 27.8 |  | Predicted protein |  |
| Wild-collected | 26.5 |  | Putative uncharacterized protein |  |
| Engorged female | 25.8 | G8946 | Immunoglobulin-binding protein |  |
| Wild-collected | 25.8 |  | *No annotation* |  |
| Wild-collected | 22.5 | G1015 | 60s ribosomal protein l15 |  |
| Wild-collected | 21.0 | G4548 | Atp-binding cassette sub-family F member |  |
| Wild-collected | 21.0 | G85 | Myosin-2 heavy chain |  |
| Wild-collected | 21.0 |  | Secreted protein |  |
| Wild-collected | 21.0 |  | Neurofilament medium polypeptide |  |
| Engorged female | 20.7 |  | *No annotation* |  |
| Engorged female | 20.7 | G1176 | Nymphal histamine-binding protein B |  |
| Larvae | 20.7 |  | *No annotation* |  |
| Larvae | 20.7 | G19488 | Secreted protein |  |
| Wild-collected | 20.7 | G4200 | Adp-ribosylation factor-like protein |  |
| Wild-collected | 20.7 |  | *No annotation* |  |
| Wild-collected | 20.7 | G1659 | Ubiquinol-cytochrome c reductase complex kda protein |  |
| Wild-collected | 20.7 |  | Glycine-rich secreted protein |  |
| Wild-collected | 20.7 |  | Putative senescence-associated protein |  |
| Wild-collected | 20.7 | G229 | *Tribolium castaneum* heat shock protein |  |
| Wild-collected/adult male | 19.8 |  | *No annotation* |  |
| Wild-collected | 19.0 |  | *No annotation* |  |
| Wild-collected | 19.0 |  | Ribosomal protein L35a |  |
| Wild-collected | 19.0 | G4993 | Hypothetical protein |  |
| **Supplemental Table 6: Identification of the 35 most abundant arthropod gene families in the *Amblyomma americanum* EST dataset.** ARP2 IDs and annotations are provided. Numbers indicate gene copy number found for the *A. americanum* ESTs (*Aam*) and for *I. scapularis* (*Isc)* as reported by eugenes/Arthropods. Gene families are ranked in order of decreasing copy number in *A. americanum.* The column Insecta represents the average copy number for each gene family across the 12 insect species included in eugenes/Arthropods. Annotations were obtained from eugenes/Arthropods. | | | | |
| \| **ARP2_ID** \| ***Aam*** \| ***Isc*** \| **Insecta** \| **Annotation** \| \| --- \| --- \| --- \| --- \| --- \| \| ARP2_G20 \| 35 \| 74 \| 2.9 \| sulfotransferase sult; bile salt sulfotransferase; hypothetical protein \| \| ARP2_G266 \| 16 \| 1 \| 3.0 \| conserved hypothetical protein \| \| ARP2_G19445 \| 12 \| 3 \| 0.0 \| secreted protein \| \| ARP2_G346 \| 12 \| 19 \| 0.3 \| cytochrome p450; cyp6g1; cyp9h1 \| \| ARP2_G85 \| 12 \| 1 \| 2.1 \| myosin-2 heavy chain \| \| ARP2_G9198 \| 12 \| 1 \| 0.3 \| hypothetical protein; serine protease inhibitor \| \| ARP2_G1422 \| 11 \| 1 \| 0.8 \| hypothetical protein; transposase; centromere protein b \| \| ARP2_G2473 \| 11 \| 16 \| 0.0 \| hypothetical protein; traf6; secreted protein \| \| ARP2_G8937 \| 11 \| 1 \| 0.6 \| Putative 115 kDa protein in type-1 retrotransposable element R1DM \| \| ARP2_G10601 \| 9 \| 6 \| 0.0 \| hypothetical protein; phosphatidylinositol transfer protein sec14; cral/trio domain-containing protein \| \| ARP2_G1176 \| 9 \| 19 \| 0.0 \| nymphal histamine binding protein b \| \| ARP2_G11892 \| 8 \| 5 \| 0.0 \| hebreain \| \| ARP2_G2475 \| 8 \| 15 \| 0.1 \| hypothetical protein; a disintegrin and metalloproteinase with thrombospondin motifs like; metalloprotease \| \| ARP2_G374 \| 8 \| 1 \| 2.5 \| intron-specific reverse transcriptase \| \| ARP2_G576 \| 8 \| 24 \| 0.1 \| scavenger receptor cysteine-rich protein; cysteine-rich venom protein; von willebrand factor \| \| ARP2_G7797 \| 8 \| 10 \| 0.0 \| secreted protein \| \| ARP2_G9664 \| 8 \| 3 \| 0.3 \| conserved hypothetical protein \| \| ARP2_G125 \| 7 \| 44 \| 0.6 \| elongation of very long chain fatty acids protein; elongase; hypothetical protein \| \| ARP2_G135 \| 7 \| 19 \| 3.0 \| serine protease inhibitor serpin; serine protease inhibitor; leukocyte elastase inhibitor \| \| ARP2_G14067 \| 7 \| 4 \| 0.0 \| RNAse h \| \| ARP2_G31 \| 7 \| 5 \| 8.3 \| cell division protein ftsj \| \| ARP2_G430 \| 7 \| 29 \| 0.0 \| hypothetical protein \| \| ARP2_G4941 \| 7 \| 14 \| 0.0 \| cystatin \| \| ARP2_G499 \| 7 \| 1 \| 1.8 \| ubiquitin/40s ribosomal protein s27a fusion \| \| ARP2_G10595 \| 6 \| 6 \| 0.0 \| hypothetical protein; btb/poz domain-containing protein; mgc154338 protein \| \| ARP2_G1446 \| 6 \| 18 \| 0.0 \| cytochrome p450 \| \| ARP2_G19437 \| 6 \| 3 \| 0.0 \| hypothetical protein \| \| ARP2_G240 \| 6 \| 1 \| 1.9 \| elongation factor-1 alpha; translation elongation factor ef-1 alpha/tu \| \| ARP2_G27748 \| 6 \| 2 \| 0.0 \| hypothetical protein; pogo family transposase \| \| ARP2_G27785 \| 6 \| 2 \| 0.0 \| hypothetical protein \| \| ARP2_G41 \| 6 \| 1 \| 5.2 \| actin-87e \| \| ARP2_G44 \| 6 \| 5 \| 6.5 \| heat shock kda protein cognate \| \| ARP2_G4943 \| 6 \| 14 \| 0.0 \| secreted salivary gland peptide \| \| ARP2_G55 \| 6 \| 91 \| 0.0 \| monocarboxylate transporter; out; monocarboxylate transporter 1 \| \| ARP2_G8895 \| 6 \| 1 \| 0.5 \| conserved hypothetical protein \| | | | | |

| **Supplemental Table 7: Identification of ARP2 gene families exclusive to non-insect arthropod species.** Non-insect species included in the ARP2 database are *A. americanum*, *I. scapularis,* and *D. pulex*. Columns of numbers indicate number of genes per gene family per species. Abbreviations correspond to non-insect taxa: *Aam (A. americanum)* from the EST database*; Isc (I. scapularis)* and *Dpu (D. pulex)* from eugenes/Arthropods*.* Annotation obtained from eugenes/Arthropods. |
| --- |
| \| **ARP2_ID** \| ***Aam*** \| ***Isc*** \| ***Dpu*** \| **Annotation** \| \| --- \| --- \| --- \| --- \| --- \| \| ARP2_G25720 \| 1 \| 1 \| 1 \| 30s ribosomal protein s12 \| \| ARP2_G24626 \| 1 \| 1 \| 1 \| 3-hydroxyanthranilate \| \| ARP2_G12099 \| 1 \| 1 \| 1 \| 40s ribosomal protein s11; tetrahymena ribosomal protein s17 containing protein \| \| ARP2_G25377 \| 1 \| 1 \| 1 \| 50s ribosomal protein l1p; 50s ribosomal protein l1 \| \| ARP2_G25965 \| 2 \| 1 \| 1 \| calponin \| \| ARP2_G25452 \| 1 \| 1 \| 1 \| cathepsin c \| \| ARP2_G25247 \| 2 \| 1 \| 1 \| chaperonin complex component \| \| ARP2_G25329 \| 1 \| 1 \| 1 \| chorismate synthase \| \| ARP2_G24647 \| 1 \| 1 \| 1 \| chromosome 7 scaf14601 \| \| ARP2_G1169 \| 1 \| 1 \| 18 \| conserved hypothetical protein \| \| ARP2_G1847 \| 2 \| 1 \| 16 \| conserved hypothetical protein \| \| ARP2_G8338 \| 1 \| 8 \| 1 \| cytochrome p450 \| \| ARP2_G25854 \| 1 \| 1 \| 1 \| dehydrogenase \| \| ARP2_G24880 \| 1 \| 1 \| 1 \| ectonucleoside triphosphate diphosphohydrolase; ectonucleoside triphosphate diphosphohydrolase 5 precursor \| \| ARP2_G25625 \| 1 \| 1 \| 1 \| endoplasmic reticulum-golgi intermediate compartment protein \| \| ARP2_G24725 \| 1 \| 1 \| 1 \| engulfment and cell motility \| \| ARP2_G25061 \| 1 \| 1 \| 1 \| f-box and wd domain protein \| \| ARP2_G16669 \| 1 \| 1 \| 2 \| f-box/leucine rich repeat protein \| \| ARP2_G25612 \| 2 \| 1 \| 1 \| glucose-6-phosphate translocase \| \| ARP2_G10424 \| 1 \| 1 \| 5 \| glutathione s-transferase;; glutathione s-transferase ec class-sigma \| \| ARP2_G17107 \| 1 \| 1 \| 1 \| glyoxalase \| \| ARP2_G25522 \| 1 \| 1 \| 1 \| gtp-binding protein enga \| \| ARP2_G17075 \| 1 \| 1 \| 2 \| hypothetical protein \| \| ARP2_G25462 \| 3 \| 1 \| 1 \| hypothetical protein \| \| ARP2_G25549 \| 2 \| 1 \| 1 \| hypothetical protein \| \| ARP2_G24879 \| 1 \| 1 \| 1 \| hypothetical protein \| \| ARP2_G25045 \| 1 \| 1 \| 1 \| hypothetical protein \| \| ARP2_G25132 \| 1 \| 1 \| 1 \| hypothetical protein \| \| ARP2_G25399 \| 1 \| 1 \| 1 \| hypothetical protein \| \| ARP2_G25441 \| 1 \| 1 \| 1 \| hypothetical protein \| \| ARP2_G25618 \| 1 \| 1 \| 1 \| hypothetical protein \| \| ARP2_G25726 \| 1 \| 1 \| 1 \| hypothetical protein \| \| ARP2_G25739 \| 1 \| 1 \| 1 \| hypothetical protein \| \| ARP2_G25903 \| 1 \| 1 \| 1 \| hypothetical protein \| \| ARP2_G25968 \| 1 \| 1 \| 1 \| hypothetical protein \| \| ARP2_G8889 \| 1 \| 1 \| 7 \| hypothetical protein \| \| ARP2_G25406 \| 1 \| 1 \| 1 \| kinesin-like protein kif1b; kinesin heavy chain; hypothetical protein \| \| ARP2_G9594 \| 2 \| 5 \| 2 \| membrane glycoprotein lig-1 \| \| ARP2_G16859 \| 1 \| 1 \| 2 \| methylmalonic aciduria type a protein \| \| ARP2_G16846 \| 1 \| 2 \| 1 \| ornithine aminotransferase \| \| ARP2_G24849 \| 1 \| 1 \| 1 \| otu domain-containing protein \| \| ARP2_G24911 \| 1 \| 1 \| 1 \| polyribonucleotide nucleotidyltransferase \| \| ARP2_G25977 \| 1 \| 1 \| 1 \| proteasome assembly chaperone \| \| ARP2_G25060 \| 1 \| 1 \| 1 \| protein tex \| \| ARP2_G9547 \| 1 \| 1 \| 6 \| receptor-transporting protein \| \| ARP2_G16772 \| 1 \| 1 \| 2 \| ribosomal rna adenine dimethylase; dimethyladenosine transferase \| \| ARP2_G25674 \| 1 \| 1 \| 1 \| riken cdna gene \| \| ARP2_G17066 \| 1 \| 1 \| 2 \| secreted protein \| \| ARP2_G25881 \| 1 \| 1 \| 1 \| secreted salivary gland peptide \| \| ARP2_G16844 \| 1 \| 1 \| 2 \| sodium-bile acid cotransporter \| \| ARP2_G9606 \| 1 \| 6 \| 1 \| transporter \| \| ARP2_G25920 \| 1 \| 1 \| 1 \| xtp3-transactivated protein a protein \| |
