# Supplementary material for: Why so many unknown genes? Partitioning orphans from a representative transcriptome of the lone star tick Amblyomma americanum
Source: BMC Genomics. 2013 Feb 27;14:135. doi: 10.1186/1471-2164-14-135 (PMC3616916; doi:10.1186/1471-2164-14-135)
Supplement: Additional file 2 — Supplemental Text. Includes discussions of the A. americanum EST dataset deconstructed according to life stage, as well as additional annotation results. [file 1471-2164-14-135-S2.docx]

SUPPLEMENTAL TEXT

***Enrichment of ESTs across libraries***

The *A. americanum* EST library was constructed from six individual libraries representing facility-reared larvae, nymphs, adult males, adult females and engorged females, as well as wild-collected adult ticks. Normalization efficiently removed highly similar expressed sequences. When sufficiently divergent, however, similar sequences from the same individual library were retained and assembled into a contig along with similar sequences from other individual libraries. We investigated biased expression according to life stage and environment within the 2,730 contigs. To do so, we compared the proportion of ESTs from each individual library within a single contig. Specifically, enrichment of an individual library for particular gene families was assessed using Chi-square tests to compare the divergence between the observed distribution of assembled sequences within a contig and the expected distribution if representation within a contig was equivalent across libraries (i.e. the total number of sequences in a contig divided by six). A significant deviation from expectation indicates that the majority of sequences in a contig are derived from one individual library, suggesting that the corresponding transcript is expressed preferentially in that life stage or population. GenBank sequences were not included in this analysis.

A total of 31 contigs were identified as being significantly enriched in sequences from one or two individual libraries, as defined by a χ^2^ cut-off of 18.47 and a p-value ≤0.001 (Suppl. Table 5). Of the 31 significantly enriched contigs, 21 were enriched by the wild-collected library, meaning that, at minimum, 35% of sequences assembled to form each of these contigs were derived from the wild-type library. Additionally, three of the 31 contigs were enriched by the larval library, four by the engorged female library, and three by both the wild-collected and adult male libraries.

The proportion of total sequences in assembled contigs that were assembled into enriched contigs was significantly divergent across individual libraries (χ^2^=168.08, df=5, p<0.001). Of the 1,466 sequences derived from the wild-collected library that were assembled into contigs, 10.8% were assembled into enriched contigs, compared to a range of 0.5% for the nymph library to 6.0% for the adult male library. Additionally, of all sequences that were assembled into enriched contigs, 53.6% were derived from the wild-collected library. This high representation is not an artifact of over-sampling of the wild-collected library (Suppl. Table 1). Of all sequences assembled into contigs, only 25.3% were derived from the wild-collected library, which is well below the representation of the wild-collected library in enriched contigs.

A majority of the 31 enriched contigs (n=25, 80.6%) were either annotated directly through UniProtKB or indirectly through matching to annotated peptides of *I. scapularis* or insect reference genomes (Suppl. Table 5). Those contigs with matches to *I. scapularis* peptides were also given eugenes/Arthropods ARP2 gene family IDs [[1](#_ENREF_1)] , as introduced previously in the Methods section. Several of the contigs enriched in engorged female and larval sequences were linked to blood-feeding and immune manipulation of the host: e.g. immunoglobulin-binding protein [[2](#_ENREF_2), [3](#_ENREF_3)], nymphal histamine binding protein [[4](#_ENREF_4), [5](#_ENREF_5)], and histamine release protein [[6](#_ENREF_6), [7](#_ENREF_7)]. Interestingly, the contig with the largest enrichment score (χ^2^=168.4, p<0.0001) was derived from the engorged female library and annotated as a programmed cell death protein. This protein may be expressed by engorged females in the process of degrading internal organs to provide nutrition for egg production [[8](#_ENREF_8)]. The 21 contigs enriched in wild-collected sequences were not obviously linked to blood-feeding. Rather, they include ribosomal proteins (n=5), uncharacterized hypothetical, predicted, or putative proteins (n=3), a myosin-2 heavy chain protein, and a heat shock protein to name a few (Suppl. Table 5).

The dominance of enriched contigs by wild-collected sequences may reflect the greater genetic diversity of the wild-collected population compared to inbred, facility-reared ticks. The probability of sequences escaping normalization due to polymorphism and genetic divergence is therefore greater for the wild-collected library. This would suggest that there should be no functional bias to the ESTs identified as enriched in the wild-collected library but rather that the enriched contigs represent more variable genes. In addition, the influences of their internal microbial community and native environment are likely to be stronger, more complex, and more variable in the wild-collected population than for facility-reared ticks. This explanation predicts a functional bias of the enriched contigs towards genes associated with microbial and environmental interactions. Many of the functional annotations described here and in Supplemental Table 5 fall into the category of house-keeping genes (e.g. ribosomal proteins, heat shock proteins), although the annotations are often general or absent (e.g. putative uncharacterized protein). This currently prevents us from distinguishing between the genetic vs. environmental diversity explanations suggested here.

***Additional annotation description***

*Representation of the* Coxiella *endosymbiont*

In spite of removal of prokaryotic sequence contaminants in constructing this EST library, a total of 97 sequences matched proteins in the UniProtKB database derived from bacteria. The majority (n=83, 85.6%) were derived from gram-negative γ-proteobacteria, and 76 matched Coxiellaceae proteins (Suppl. Fig. 3a). Several were redundant such that 72 unique proteins were ultimately identified. The frequency and retention of Coxiellaceae sequences following removal of prokaryotic sequences during library construction indicate a high abundance within the tick, consistent with the *Coxiella* endosymbiont of *A. americanum* [[9-11](#_ENREF_9)].

The paucity of genomic data for this and other endosymbiotic *Coxiella* species may explain why 72 of 76 sequences matched proteins derived from *C. burnetti*, the agent of Q-fever, for which a complete genome sequence exists. These sequences are unlikely to represent *C. burnetti,* since *A. americanum* is not a vector of this pathogen [[12-14](#_ENREF_12)]. Accordingly, phylogenetic reconstruction shows clustering of *A. americanum* sequence with the *Coxiella* endosymbiont of *A. americanum* rather than with *C. burnetii* (Suppl. Fig. 4). A Neighbor-Joining tree was reconstructed of *rpsG* genes from the *A. americanum* EST library (N=1) (seq_B02_wt_6d_007.abi), *C. burnetii* (N=6), the *Coxiella* endosymbiont of *A. americanum* (N=1), and four other γ-proteobacteria using the Jukes-Cantor model with 70% similarity.

Classification of these *Coxiella* sequences as endosymbiont sequences is further supported by the distribution of Coxiellaceae sequences across individual EST libraries. Endosymbiotic bacteria of blood-feeding organisms are frequently over-represented in adult female hosts. Here, Coxiellaceae sequences were primarily derived from the adult female (n=36, 37.5%) and wild-collected (n=37, 38.5%) libraries, the latter being composed of 50% females (Suppl. Fig. 3b). Only two Coxielliaceae sequences (2.08%) were derived from the engorged female library, although *Coxiella* endosymbionts are readily detectable in the reproductive tissues of engorged females using fluorescent in situ hybrization and targeted diagnostic PCR assays [[11](#_ENREF_11)]. Our findings are consistent with recent quantitative PCR results suggesting that the *Coxiella* endosymbiont is in relatively low abundance in eggs and early life stages but amplifies 20-60 fold in the questing female tick (Clay et al. unpublished data).

*Arthropod gene families in* A. americanum

Conservation across the arthropod phylum was further investigated through eugenes/Arthropods ARP2 IDs [[1](#_ENREF_1)]. By ranking ARP2 gene families according to abundance in the *A. americanum* EST library, several gene families were identified as being highly represented. The 35 most highly represented are reported in Supplemental Table 6. The most highly represented gene family (N=35) is annotated as a hypothetical bile salt sulfotransferase, which is also significantly over-represented in *I. scapularis* relative to other species in the ARP2 dataset [[15](#_ENREF_15)]. Many functions may be ascribed to sulfotransferases, though Pichu et al. [[16](#_ENREF_16)] propose a specific role for sulfotransferases in modulating tick salivation and homeostasis during blood-feeding. Several other highly represented gene families may also contribute to blood-feeding: nine nymphal histamine-binding b proteins [[4](#_ENREF_4), [5](#_ENREF_5)], eight hebraein proteins [[17](#_ENREF_17)], 19 serine protease inhibitors [[18](#_ENREF_18), [19](#_ENREF_19)], seven cystatins [[20](#_ENREF_20)], eight metalloproteases [[21](#_ENREF_21)]; eight von Willebrand factors [[22](#_ENREF_22), [23](#_ENREF_23)] and six secreted salivary gland peptides. Other notable gene families, whose functional relevance is as yet undefined, include 12 myosin-2 heavy chain proteins and 18 cytochrome P450-related proteins, heme-based enzymes that perform a wide variety of oxidation reactions on low molecular weight substrates, often involved in detoxification.

We also investigated gene families exclusive to chelicerates (*I. scapularis* and *A. americanum*) and crustaceans (*D. pulex*). These likely represent gene families lost in the class Insecta. Among all ARP2 gene families, 248 are exclusive to *I. scapularis* and *D. pulex.* A subset of these (N=52) were also identified in the *A. americanum* EST library (Suppl. Table 7). Among these gene families, 18 were described as hypothetical proteins, four as ribosomal proteins, one as a cytochrome P450, one as an uncharacterized secreted protein, and one as a secreted salivary gland peptide. The absence of specific functional information in this group of gene families probably reflects the dearth of research focused on arthropod gene families outside the Insecta.

**References**

1. Gilbert D: **OrthoMCL clustering among 14 arthropod proteins (ARP2)**. In*.*, Dec. 2009 edn; Dec. 2009.

2. Wang H, Nuttall PA: **Excretion of host immunoglobulin in tick saliva and detection of IgG-binding proteins in tick hemolymph and salivary glands**. *Parasitology* 1994, **109**:525-530.

3. Wang H, Nuttall PA: **Immunoglobulin-binding proteins in ticks: new target for vaccine development against a blood-feeding parasite**. *Cellular and Molecular Life Sciences* 1999, **56**(3-4):286-295.

4. Aljamali MN, Bior AD, Sauer JR, Essenberg RC: **RNA interference in ticks: a study using histamine binding protein dsRNA in the female tick *Amblyomma americanum***. *Insect Mol Biol* 2003, **12**(3):299-305.

5. Paesen GC, Adams PL, Harlos K, Nuttall PA, Stuart DI: **Tick histamine-binding proteins: Isolation, cloning, and three-dimensional structure**. *Mol Cell* 1999, **3**(5):661-671.

6. Dai J, Narasimhan S, Zhang L, Liu L, Wang P, Fikrig E: **Tick histamine release factor is critical for *Ixodes scapularis* engorgement and transmission of the lyme disease agent**. *PLoS pathogens* 2010, **6**(11):e1001205.

7. Mulenga A, Macaluso KR, Simser JA, Azad AF: **The American dog tick, *Dermacentor variabilis*, encodes a functional histamine release factor homolog**. *Insect Biochem Mol Biol* 2003, **33**(9):911-919.

8. Aljamali MN, Ramakrishnan VG, Weng H, Tucker JS, Sauer JR, Essenberg RC: **Microarray analysis of gene expression changes in feeding female and male lone star ticks, *Amblyomma americanum* (L)**. *Arch Insect Biochem Physiol* 2009, **71**(4):236-253.

9. Clay K, Klyachko O, Grindle N, Civitello D, Oleske D, Fuqua C: **Microbial communities and interactions in the lone star tick, *Amblyomma americanum***. *Mol Ecol* 2008, **17**(19):4371-4381.

10. Jasinskas A, Zhong J, Barbour A: **Highly prevalent *Coxiella* sp. bacterium in the tick vector *Amblyomma americanum***. *Applied and Environmental Microbiology* 2007, **73**(1):334-336.

11. Klyachko O, Stein BD, Grindle N, Clay K, Fuqua C: **Localization and visualization of a *Coxiella*-type symbiont within the lone star tick, *Amblyomma americanum***. *Appl Environ Microbiol* 2007, **73**(20):6584-6594.

12. Childs J, Paddock C: **The ascendancy of *Amblyomma americanum* as a vector of pathogens affecting humans in the United States**. *Ann Rev Entomol* 2003, **48**:307-337.

13. Thompson H, Dennis D, Dasch GA: **Q fever**. In: *Tick-Borne Diseases of Humans.* Edited by Goodman J, Dennis D, Sonenshine DE. Washington, DC: ASM Press; 2005: 328-342.

14. Goddard J, Varela-Stokes A: **Role of the lone star tick, *Amblyomma americanum* (L.), in human and animals diseases**. *Veterinary Parasitology* 2009, **160**:1-12.

15. Gilbert D: **Ixodes orthology gene groups with over-abundance compared to insects**. In*.* Edited by Gilbert D: <http://arthropods.eugenes.org/arthropods/> 2009.

16. Pichu S, Yalcin E, Ribeiro J, King R, Mather T: **Molecular characterization of novel sulfotransferases from the tick, Ixodes scapularis**. *BMC Biochemistry* 2011, **12**(1):32.

17. Lai R, Takeuchi H, Lomas LO, Jonczy J, Rigden DJ, Rees HH, Turner PC: **A new type of antimicrobial protein with multiple histidines from the hard tick, Amblyomma hebraeum**. *FASEB Journal* 2004, **18**(10).

18. Chalaire KC, Kim TK, Garcia-Rodriguez H, Mulenga A: **Amblyomma americanum (L.) (Acari: Ixodidae) tick salivary gland serine protease inhibitor (serpin) 6 is secreted into tick saliva during tick feeding**. *J Exp Biol* 2011, **214**(4):665-673.

19. Mulenga A, Sugino M, Nakajima M, Sugimoto C, Onuma M: **Tick-encoded serine proteinase inhibitors (Serpins); Potential target antigens for tick vaccine development**. *J Vet Med Sci* 2001, **63**(10):1063-1069.

20. Karim S, Miller NJ, Valenzuela J, Sauer JR, Mather TN: **RNAi-mediated gene silencing to assess the role of synaptobrevin and cystatin in tick blood feeding**. *Biochem Biophys Res Commun* 2005, **334**(4):1336-1342.

21. Decrem Y, Mariller M, Lahaye K, Blasioli V, Beaufays J, Boudjeltia KZ, Vanhaeverbeek M, Cerutti M, Brossard M, Vanhamme L *et al*: **The impact of gene knock-down and vaccination against salivary metalloproteases on blood feeding and egg laying by Ixodes ricinus**. *International Journal for Parasitology* 2008, **38**(5):549-560.

22. Sadler JE: **von Willebrand factor**. *J Biol Chem* 1991, **266**(34):22777-22780.

23. von Willebrand E: **Hereditär pseudohemofili**. *Fin Läkaresällsk Handl* 1926, **68**:87-112.
