# Supplementary material for: Why so many unknown genes? Partitioning orphans from a representative transcriptome of the lone star tick Amblyomma americanum
Source: BMC Genomics. 2013 Feb 27;14:135. doi: 10.1186/1471-2164-14-135 (PMC3616916; doi:10.1186/1471-2164-14-135)
Supplement: Additional file 3 — Figures. Figures S1 through S4 and associated legends. [file 1471-2164-14-135-S3.pdf]

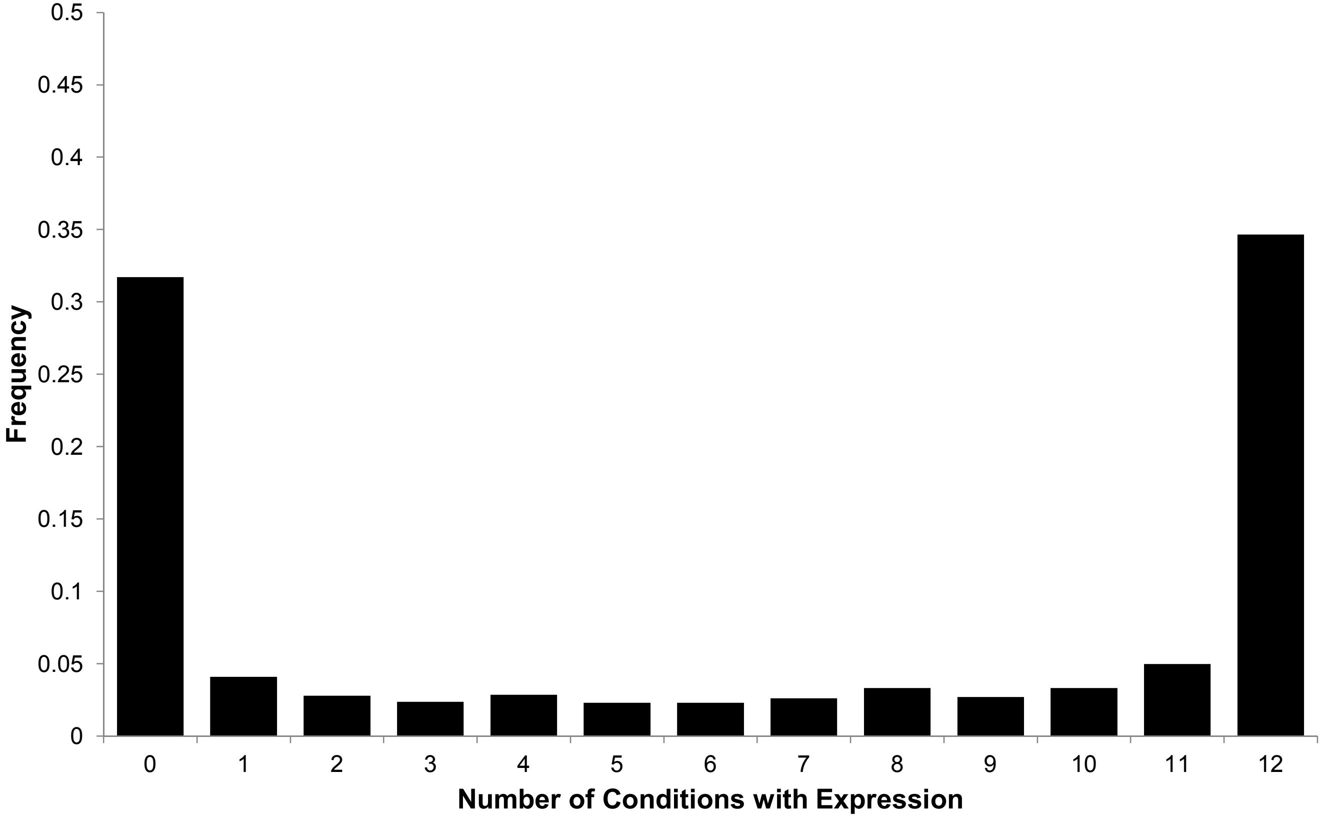

Supplemental Figure 1: Frequency distribution of expression of 13,962 microarray probes in 12 conditions. Microarray probes were derived from the *Amblyomma americanum* transcriptome. Histogram indicates the proportion of total probes that had detectable levels of transcription in zero to 12 of the conditions, based upon a 0.5% false discovery rate. Sequences expressed in zero or one conditions (N=5000) were classified as non-functional.

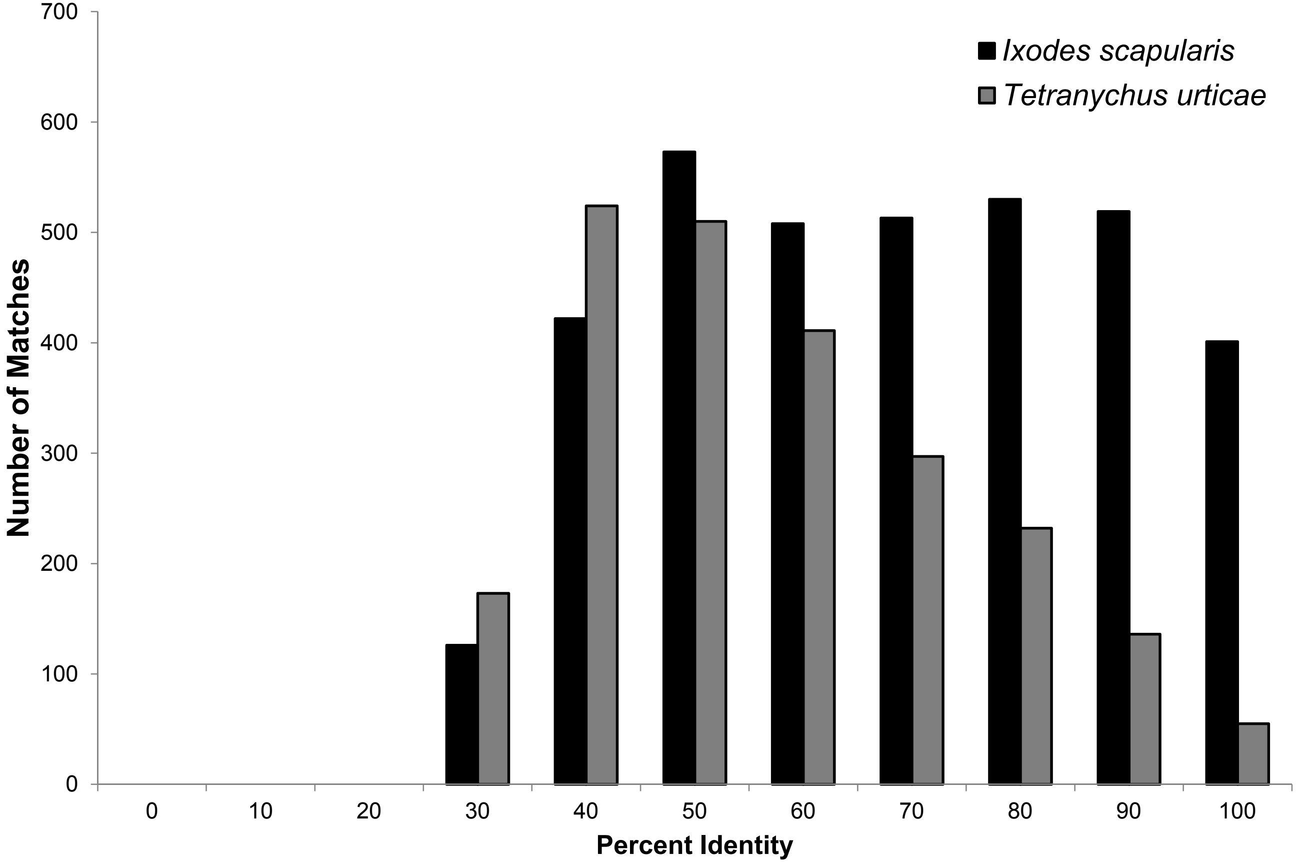

Supplemental Figure 2: Frequency distribution of the percent identities of matches of *Amblyomma americanum* ESTs with representative *Ixodes scapularis* and *Tetranychus urticae* datasets. Matches were obtained by BLAST searches of *A. americanum* ESTs against the *I. scapularis* predicted peptides (available at VectorBase) or against the *T. urticae* predicted peptides (availabled at BOGAS). An e-value and match length cutoff of 1e-5 and 33 amino acids, respectively, were applied for all BLAST results. A total of 3,592 matches were obtained against *I. scapularis* and 2,338 against *T. urticae*.

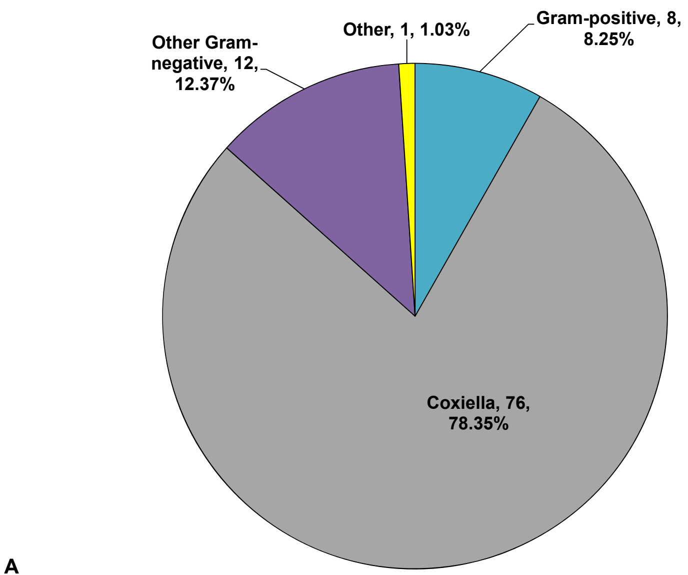

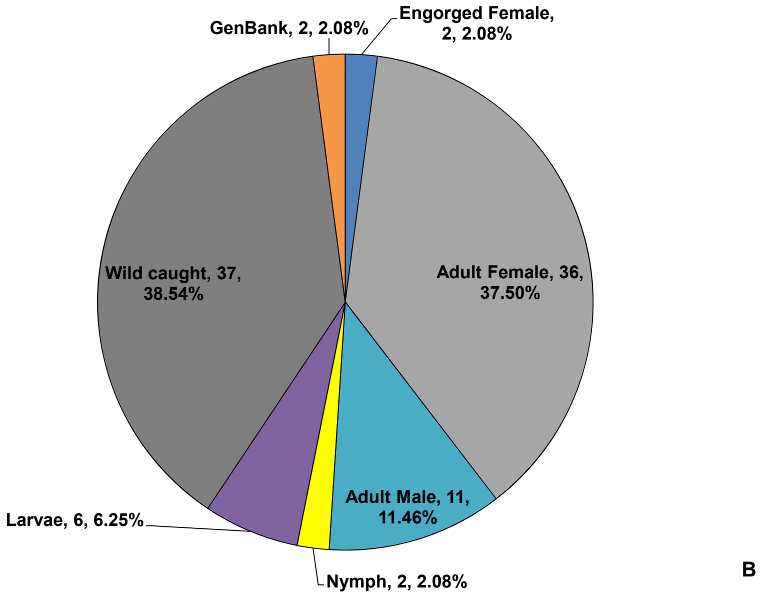

Supplemental Figure 3: Description of the *Amblyomma americanum* microbial community. (A) The taxonomic distribution of 97 UniProtKB microbial protein matches. (B) The distribution across individual tick libraries of 76 sequences (13 contigs, 63 singletons) matching Coxielliaceae proteins.

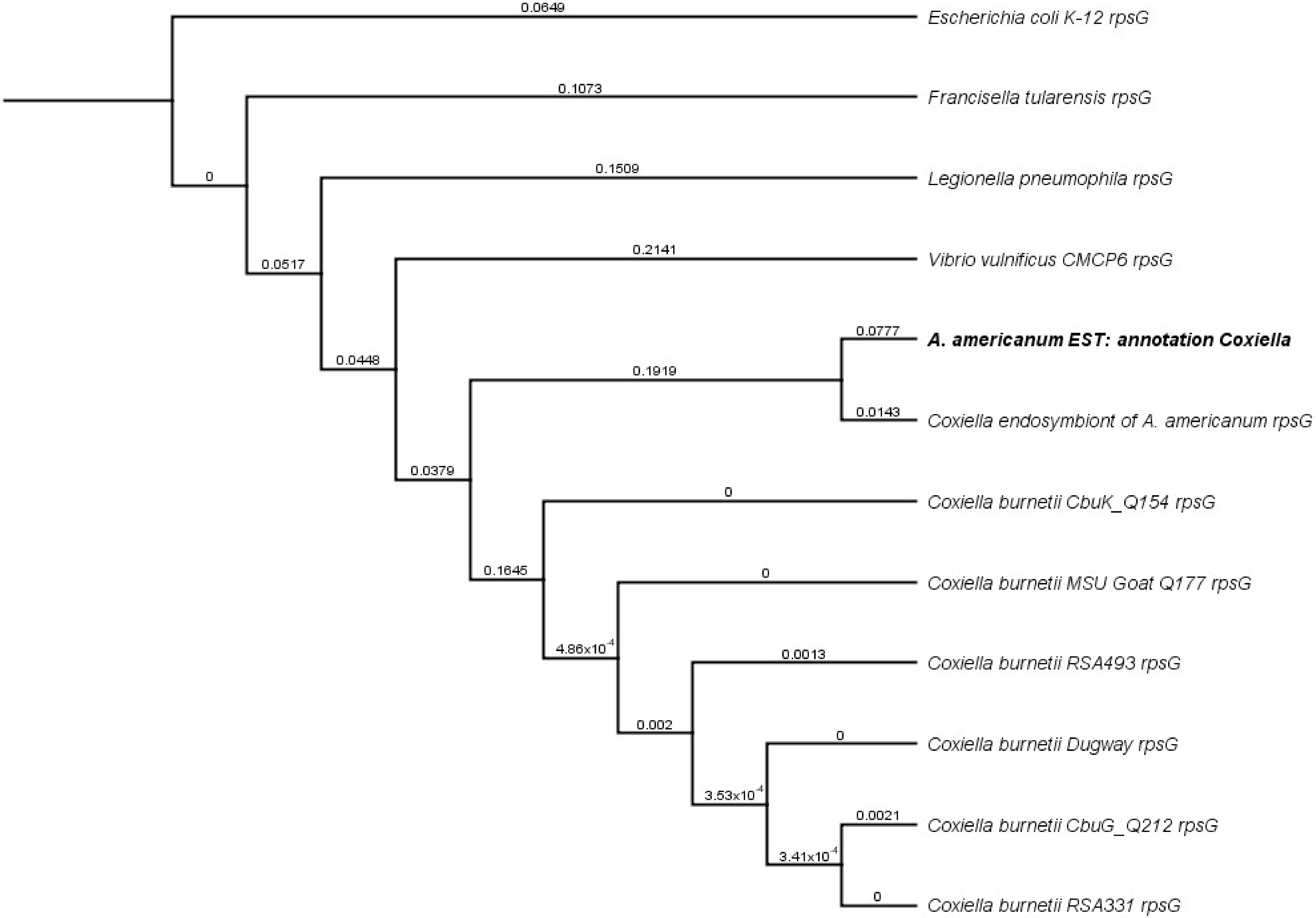

Supplemental Figure 4: Phylogenetic reconstruction of *Amblyomma americanum Coxiella* EST sequences. Representative *Coxiella* EST sequence derived from *A. americanum* transcriptome groups with sequences of the *A. americanum Coxiella* endosymbiont rather than with *C. burnetii* sequences. This reconstruction includes six *C. burnetii* rpsG sequences, one rpsG sequence from the *Coxiella* endosymbiont of *A. americanum,* and rpsG sequences from four other gamme-proteobacteria. *A. americanum* EST annotation *Coxiella* (bolded) is a *Coxiella* endosymbiont EST generated by this study (seq_B02_wt_63_007.abi). Phylogenetic reconstruction is a Neighbor-Joining tree employing the Jukes-Cantor Genetic Distance Model set at 70% similarity. Branch labels indicate calculated substitutions per site in the aligned sequences.
